# Use of a promiscuous, constitutively-active bacterial enhancer-binding protein to define the σ^54^ (RpoN) regulon of *Salmonella* Typhimurium LT2

**DOI:** 10.1186/1471-2164-14-602

**Published:** 2013-09-05

**Authors:** David J Samuels, Jonathan G Frye, Steffen Porwollik, Michael McClelland, Jan Mrázek, Timothy R Hoover, Anna C Karls

**Affiliations:** 1 Department of Microbiology, University of Georgia, 30602, Athens, GA, USA; 2 US Department of Agriculture, Bacterial Epidemiology and Antimicrobial Resistance Research Unit, Richard B. Russell Research Center, Agricultural Research Service, 30605, Athens, GA, USA; 3 Department of Microbiology and Molecular Genetics, University of California, 92697, Irvine, CA, USA; 4 Institute of Bioinformatics, University of Georgia, 30602, Athens, GA, USA

**Keywords:** Sigma54, RpoN, Bacterial enhancer-binding protein, Regulon, Sigma factor, *Salmonella*

## Abstract

**Background:**

Sigma54, or RpoN, is an alternative σ factor found widely in eubacteria. A significant complication in analysis of the global σ^54^ regulon in a bacterium is that the σ^54^ RNA polymerase holoenzyme requires interaction with an active bacterial enhancer-binding protein (bEBP) to initiate transcription at a σ^54^-dependent promoter. Many bacteria possess multiple bEBPs, which are activated by diverse environmental stimuli. In this work, we assess the ability of a promiscuous, constitutively-active bEBP—the AAA+ ATPase domain of DctD from *Sinorhizobium meliloti*—to activate transcription from all σ^54^-dependent promoters for the characterization of the σ^54^ regulon of *Salmonella* Typhimurium LT2.

**Results:**

The AAA+ ATPase domain of DctD was able to drive transcription from nearly all previously characterized or predicted σ^54^-dependent promoters in *Salmonella* under a single condition. These promoters are controlled by a variety of native activators and, under the condition tested, are not transcribed in the absence of the DctD AAA+ ATPase domain. We also identified a novel σ^54^-dependent promoter upstream of STM2939, a homolog of the *cas1* component of a CRISPR system. ChIP-chip analysis revealed at least 70 σ^54^ binding sites in the chromosome, of which 58% are located within coding sequences. Promoter-*lacZ* fusions with selected intragenic σ^54^ binding sites suggest that many of these sites are capable of functioning as σ^54^-dependent promoters.

**Conclusion:**

Since the DctD AAA+ ATPase domain proved effective in activating transcription from the diverse σ^54^-dependent promoters of the *S*. Typhimurium LT2 σ^54^ regulon under a single growth condition, this approach is likely to be valuable for examining σ^54^ regulons in other bacterial species. The *S*. Typhimurium σ^54^ regulon included a high number of intragenic σ^54^ binding sites/promoters, suggesting that σ^54^ may have multiple regulatory roles beyond the initiation of transcription at the start of an operon.

## Background

Transcription in eubacteria is mediated by the RNA polymerase holoenzyme (Eσ), which has five constant subunits (α_2_ββ’ω) and a variable subunit (σ). The constant subunits constitute the RNA polymerase core (RNAP), which has the polymerization activity; the σ subunit determines promoter recognition and functions in the Eσ-promoter transition from closed complex to open complex (isomerization). The primary σ factor in a bacterium, such as σ^70^ in *Escherichia coli*, controls transcription of most housekeeping genes in the cell; alternative sigma factors have specialized regulons that function in the response to environmental stressors or morphological changes, or in developmental systems (for review see [1]). In many bacteria the alternative σ factor σ^54^ (also called RpoN or NtrA) has unusually diverse regulons, with genes that function in a variety of cellular processes, including flagellar biogenesis, response to nitrogen starvation, transport and metabolism of carbon substrates, and tolerance to heavy metals [2-6].

**Figure 1:**
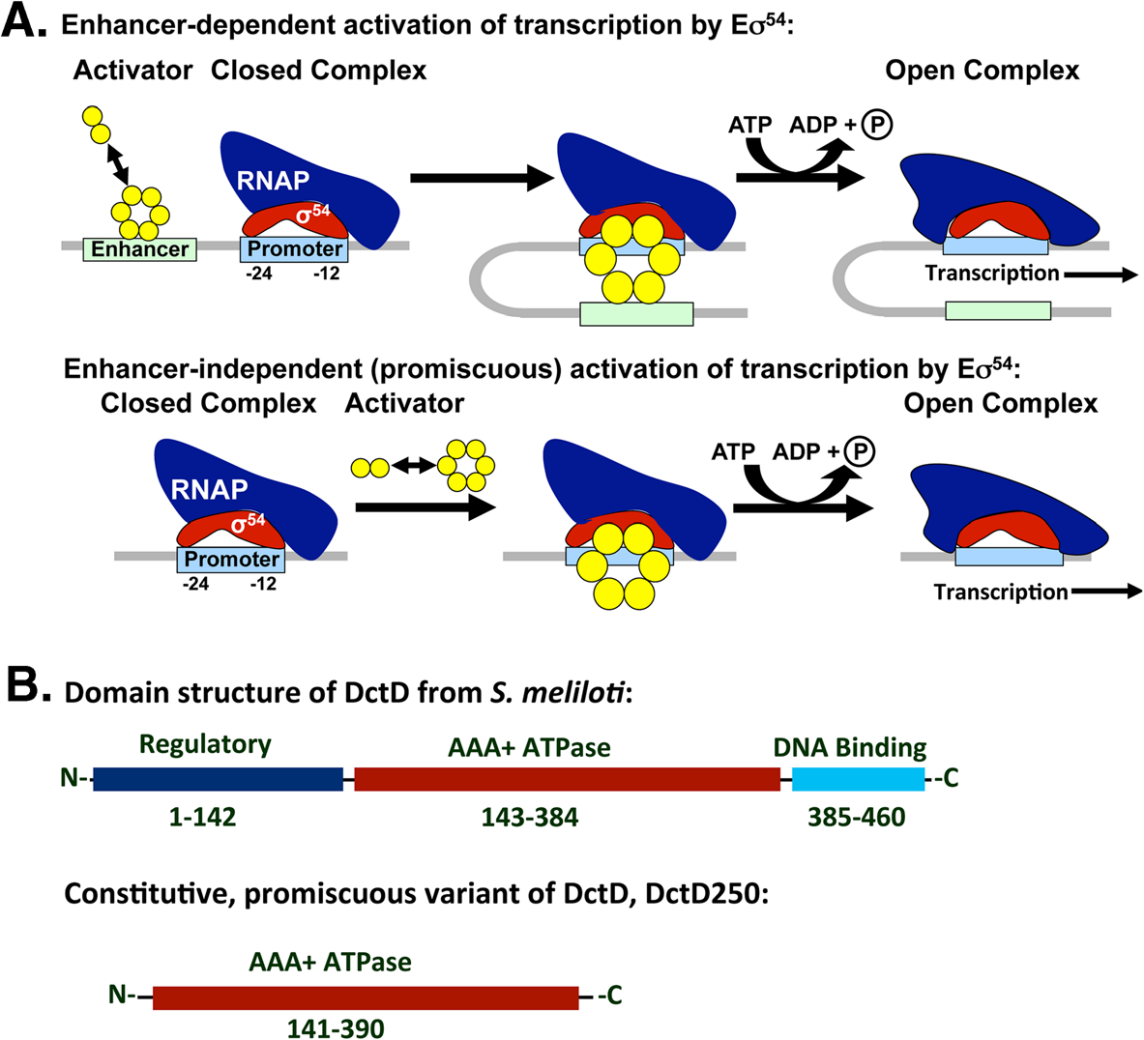
**Activation of σ**^**54**^**-dependent transcription and activator structure. A)** σ^54^ (red subunit) directs binding of the RNA polymerase (dark blue subunit) holoenzyme (Eσ^54^) to the -12, -24 promoter elements (light blue box). This closed complex is stable and cannot transition to open complex. In response to an environmental or cellular signal, the activator (bEBP; yellow dimers) oligomerizes. For most bEBPs, the oligomer binds to an enhancer (green box) 80 to 150 bp upstream of the promoter and DNA looping brings the activator in contact with σ^54^ in the Eσ^54^ closed complex. Hydrolysis of ATP by bEBP causes remodeling of Eσ^54^, which leads to open complex formation and transcription. There are a few bacteria with bEBPs that are missing the DNA binding domain; after oligomerization, these activators can bind to Eσ^54^ in closed complex with any promoter to stimulate open complex formation (promiscuous activation). **B)** The domain structure for the *Sinorhizobium meliloti* bEBP, DctD, is typical of most bEBPs. The amino-terminal regulatory domain (dark blue box) inhibits assembly of the bEBP oligomer until it interacts with an activation signal; the AAA+ ATPase domain (red box) mediates ATP binding and hydrolysis, as well as the protein-protein interactions between bEBPs (oligomerization) and between bEBP and σ^54^; the carboxyl-terminal DNA binding domain (aqua box) contains a helix-turn-helix motif for binding the enhancer. The truncated DctD variant, DctD250, is missing the regulatory and DNA binding domains, so that it is constitutively active and promiscuous in stimulating transcription from σ^54^-dependent promoters.

Multiple features, including protein structure, promoter consensus sequence, and mode of activation, distinguish σ^54^ from all other primary and secondary sigma factors, which constitute the σ^70^ family (reviewed in [1,7]). Although both σ^54^- and σ^70^-type sigma factors associate with the β and β’ subunits of RNAP and mediate the binding of Eσ to specific promoter sequences, σ^54^ differs extensively from σ^70^-type sigma factors in primary amino acid sequence and domain organization (reviewed in [8]). The essential promoter features for Eσ^54^ recognition and binding center around conserved GG and TGC elements at -24 and -12, respectively, relative to the transcription start site (TSS) [9], while holoenzymes with the various σ^70^-type sigma factors generally recognize and bind promoter elements at -35 and -10 with the consensus sequences TTGACA and TATAAT, respectively (reviewed in [1]). Perhaps the most important feature of Eσ^54^ that differs from Eσ^70^ is the isomerization process (Figure 1A). For Eσ^70^ the transition from closed complex to open complex is usually spontaneous and rapid, so regulation of transcription initiation frequently occurs at the level of closed complex formation. Initiation of transcription by Eσ^54^ more closely resembles eukaryotic Pol II systems in that Eσ^54^ forms a stable closed complex that requires a bacterial enhancer-binding protein (bEBP) and ATP hydrolysis for isomerization to open complex (reviewed in [10]). The bEBPs add an additional level of complexity to the σ^54^ regulon.

bEBPs have a modular structure that is generally conserved: an N-terminal regulatory domain, a central AAA+ ATPase domain, and a C-terminal DNA binding domain (Figure 1B; reviewed in [8]). These proteins activate transcription from σ^54^-dependent promoters in three basic steps (Figure 1A). First, the bEBP receives an environmental stimulus through phosphorylation, ligand binding, or protein-protein interactions with the N-terminal regulatory domain that stimulates the bEBP to multimerize through the AAA+ ATPase domain and bind to an upstream activator sequence (UAS or enhancer) via the C-terminal DNA binding domain. The bEBP-UAS complex is then brought into contact with the Eσ^54^-promoter closed complex via a DNA looping event and interactions between highly conserved regions of the AAA+ ATPase domain of bEBP and σ^54^. Finally, ATP hydrolysis drives isomerization, allowing the initiation of transcription.

The requirement for bEBP-mediated activation of σ^54^-dependent transcription presents two problems for global analysis of a σ^54^ regulon. The first is the need for the proper environmental stimulus to activate bEBPs. Since the Eσ^54^ closed complex requires an activated bEBP, σ^54^-dependent promoters are usually transcriptionally silent in the absence of the specific stimulus for the bEBP [8]. Analysis of transcription from σ^54^-dependent promoters under any single growth condition would miss operons whose bEBPs are not activated under the condition tested. Secondly, the requirement for the UAS or enhancer by most bEBPs presents a challenge for predicting whether a Eσ^54^ binding site is functioning as a promoter or not. There is no common consensus sequence for the enhancer and their position relative to the promoter can be quite variable. For many σ^54^-dependent promoters the UAS sequence lies ~70-150 bp upstream of the promoter, but other configurations have been characterized, such as enhancers located 1.5 kb downstream of the *rocG* promoter in *Bacillus subtilis*[11] and up to 3 kb upstream of the promoter in artificial constructs of the *glnA* operon from *E*. *coli*[12]. If a σ^54^ binding site is examined for promoter activity in isolation, such as in a promoter-reporter vector, it is difficult to discern whether a site is inactive because it is not a promoter or because the enhancer was not included in the cloned sequence.

Previous studies to define the σ^54^ regulons of *Escherichia coli*[13], *Vibrio cholerae*[14] and *Geobacter sulfurreducens*[15] have recognized the limitations presented by the requirement for activated bEBPs in the characterization of the full σ^54^ regulon, even when σ^54^ is overexpressed from a heterologous promoter. Our approach to overcoming these problems in the global characterization of σ^54^ regulons in bacteria is the utilization of a constitutively-active, promiscuous bEBP, the AAA+ ATPase domain of *Sinorhizobium meliloti* DctD [16,17]. We chose to assess the efficacy of this approach in *Salmonella enterica subsp*. *enterica* serovar Typhimurium LT2 (hereafter referred to as *S*. Typhimurium LT2), a widely-used laboratory strain, because it has a moderately-sized σ^54^ regulon with 13 known or predicted bEBPs [18], providing sufficient diversity in bEBPs to test our hypothesis.

We report here that use of this constitutively-active, promiscuous bEBP in DNA microarrays and promoter function assays permitted detection of nearly all known and predicted σ^54^-dependent operons. These studies also revealed a new σ^54^-dependent promoter expressing a putative *cas1* gene in *S*. Typhimurium LT2 (STM2938). In addition, chromatin immunoprecipitation-microarray (ChIP-chip) analysis combined with bioinformatics identified 70 Eσ^54^ or σ^54^ binding sites, of which 41 appear to be within open reading frames (ORFs). This surprising number of intragenic sites suggests regulatory roles for σ^54^ or Eσ^54^ that may involve repression, transcriptional interference, or expression of cis- or trans-acting small RNA (sRNA) [19,20].

## Results and discussion

### Utility of a promiscuous, constitutive bEBP in characterizing the σ^54^ regulon

Since all known σ^54^-dependent promoters require an activated bEBP for transcription initiation, it is a challenge to find a condition under which all promoters can be detected within the σ^54^ regulon of a bacterium. In the recent mapping of the *S*. Typhimurium SL1344 transcriptome using early stationary phase cultures in rich media (Lennox broth), only one of the known or predicted σ^54^-dependent gene transcripts was detected, *pspA*[21]. The currently favored approach is over-expression of σ^54^ to facilitate detection of σ^54^-dependent promoters, which assumes a reasonable basal level of activation of the bEBPs. Using relatively low cutoffs for the fold-change (1.5- to 2-fold) in transcript levels between the σ^54^-overexpression strain and wild type or Δ*rpoN* strains, a considerable portion of the σ^54^-dependent transcriptome was defined in *Escherichia coli*[13], *Vibrio cholerae*[14] and *Geobacter sulfurreducens*[15]. However, not all previously-identified σ^54^-dependent operons were detected for *E*. *coli* and *G*. *sulfurreducens*, and evidence from the *V*. *cholera* and *G*. *sulfurreducens* studies suggests that overexpression of σ^54^ may repress expression from some σ^54^-dependent promoters and alter expression of σ^54^-independent promoters [13-15]. We hypothesize that a promiscuous and constitutive variant of the bEBP DctD from *S*. *meliloti* can activate transcription from all σ^54^-dependent promoters in *S*. Typhimurium LT2 at wild-type levels of σ^54^ under a single growth condition, thereby facilitating global characterization of the σ^54^ regulon without overexpression of σ^54^. This promiscuous and constitutive DctD variant is missing the N-terminal response regulator and C-terminal DNA binding domains, leaving only the central AAA+ ATPase domain, residues 141 to 390 of DctD and referred to hereafter as DctD250 [17]. Previous work showed that DctD250 was able to interact with Eσ^54^ in *E*. *coli* to drive transcription from the chromosomal *glnA* promoter and from the *S*. *meliloti dctA* promoter in the absence of native DctD and without an enhancer sequence [16,17].

The σ^54^-dependent promoters of *S*. Typhimurium LT2 are normally responsive to one or more of thirteen known and predicted bEBPs under various growth conditions [18], so to initially assess DctD250 activation of transcription from σ^54^-dependent promoters that respond to different bEBPs in *Salmonella*, the σ^54^-dependent promoters for the *glnKamtB* (STM0462) and *rtcBA* (STM3521) operons were introduced upstream of a promoter-less *lacZ* gene and the reporter plasmids were transformed into a derivative of *S*. Typhimurium LT2 (wild-type; WT) and WT containing the DctD250 expression plasmid (WT + DctD250) to perform β-galactosidase assays. The *glnKamtB* and *rtcBA* promoters were chosen because neither has predicted σ^70^-dependent promoters within the cloned promoter region and each is responsive to a different bEBP: NtrC for *glnKamtB*[22] and RtcR for *rtcBA*[23]. In the WT strain, the *glnKamtB* and *rtcBA* operon promoters expressed *lacZ* at very low levels; but in the presence of DctD250, *lacZ* was expressed at 150- and 16-fold higher levels, respectively (Table 1). To compare the level of expression stimulated by DctD250 to the level that is seen under physiological conditions that activate the promoter-associated bEBP, *lacZ* expression from the *glnKamtB* promoter was assayed in the WT strain in nitrogen-limiting medium, which activates NtrC. Under nitrogen-starvation conditions NtrC multimerizes, binds the enhancer in the cloned promoter region, and hydrolyzes ATP to stimulate transcription by Eσ^54^ at the *glnKamtB* promoter (see Figure 1A). In the presence of activated NtrC, the *glnKamtB* promoter expresses *lacZ* at a nearly 10-fold higher level than in the presence of DctD250. This reduced level of activation by DctD250 relative to the cognate bEBP under activation conditions is consistent with previous studies comparing the activity of truncated versions of bEBPs, which must interact with Eσ^54^ from solution, to that of the wild type bEBPs, which are directed to the target σ^54^ promoter via binding to the enhancer sequence [17,24]. The control reporter plasmids pDV6, which has the σ^70^-dependent, circle junction promoter from IS*492*[25], and the promoter-less pDS12 expressed *lacZ* at approximately the same level in WT as WT + DctD250 (Table 1). Based on these results, DctD250 activates transcription from σ^54^-dependent promoters that are normally responsive to different bEBPs under different growth conditions. Therefore, we performed DNA microarray and promoter-reporter analyses in the presence of the promiscuous, constitutive activator DctD250 to assess the efficacy of this approach in defining the σ^54^ regulon of *S*. Typhimurium LT2.

**Table 1:** **DctD250-dependent activity of predicted and potential σ**^**54**^**-dependent promoters**

		**Miller units**^**b**^	
**Promoter**^**a**^	**WT**	**WT+DctD250**	**WT-N**
***Intergenic:***			
STM0462 (*glnKamtB*)	1.1 ±0.1	180 ±22	1778 ±28
STM3521 (*rtcBA*)	14 ±6.8	220 ±55	N.D.
***Intragenic:***			
STM0699	5.3 ±1.6	56 ±6.2	N.D.
STM2430	19 ±3.8	90 ±5.5	N.D.
STM2939	2.2 ±1.4	76 ±17	N.D.
***Controls:***			
IS*492*-CJ (pDV6)	6400 ±2200	9000 ±4100	N.D.
Empty vector (pDS12)	2.0 ±0.5	8.4 ±3.5	4 ±0

^**a**^Promoters assayed in *lacZ*-reporter plasmids pDS11 and pDS12: intergenic promoters are predicted promoters for the *glnALG*, *glnKamtB* and *rtcBA* operons; intragenic promoters were identified in the ChIP-chip assay (see Results). Controls are the σ^70^-dependent, circle-junction promoter from IS*492* and the empty vector, pDS12.

^**b**^β-galactosidase assays were performed in MOPS minimal medium (WT and WT+DctD250) or in nitrogen-limiting MOPS (WT-N).

### Microarray analysis of σ^54^-dependent transcripts in *Salmonella* expressing DctD250

To determine the genes whose transcription is controlled by σ^54^ in *S*. Typhimurium LT2 we performed a microarray analysis comparing WT+DctD250 to an isogenic strain with a deletion of *rpoN* (Δ*rpoN*+DctD250). RNA collected during mid-log phase growth in nutrient medium was reverse transcribed and cDNAs from each strain were differentially labeled and applied to a complete ORF array containing all annotated open reading frames for *S*. Typhimurium LT2 [26]. Open reading frames that were transcribed in WT at a level > 3-fold higher than in the Δ*rpoN* strain, with a p-value <0.02, were considered up-regulated and, for the purpose of the initial categorization of these results, an operon was considered up-regulated if at least one gene met these criteria. In three biological replicates, the same 33 operons were up-regulated in the presence of σ^54^. The microarray results for *S*. Typhimurium LT2 genes within operons that meet the criteria for up-regulation, or that are known or predicted to be σ^54^-dependent, are shown in Table 2 and Additional file 1. Only 4 genes, STM2722, STM2724, STM2729, and STM2730, which are part of 2 operons in the Fels-2 prophage, were down-regulated >3-fold with a p-value <0.02 in the WT strain as compared to the Δ*rpoN* strain.

**Table 2:** **Microarray results for known, predicted, and novel σ**^**54**^**-dependent operons and sRNA genes of *S*. Typhimurium**

**Locus tag**^**a**^	**Gene symbol**^**b**^	**Function**	**bEBP**^**c**^	**WT/Δ*rpoN***^**d**^	**Ref.**^**e**^
***Known σ***^***54***^**- *dependent operons and sRNA genes *:**					
**STM0368-71**	*prpBCDE*	Proprionate catabolism (putative)	PrpR	**45**	[4]
**STM0830-28**	*glnHPQ*	Glutamine high-affinity transporter	NtrC	**7.1**	[27]
**STM2355**	*argT*	Lysine/arginine/ornithine transport protein	NtrC	**3.5**	[2]
STM_R0152	*glmY*	GlmY sRNA	GlrR	0.9	[28]
STM_R0167	*glmZ*	GlmZ sRNA	GlrR	1.1	[28]
**STM4007-05**	*glnALG*	Glutamine synthetase	NtrC	**48**	[29]
***Predicted σ***^***54***^**- *dependent operons *:**					
**STM0462-63**	*glnK amtB*	hypothetical protein	NtrC	**3.6**	[22]
**STM0577-72**		PTS (putative)	STM0571	**67**	[18]
**STM0649.S-53**		Hydrolase (putative)	STM0652	**11**	[18]
STM0665-62	*gltIJKL*	Glutamate/aspartate transporter	NtrC	1.8	[18,30]
STM1285-84	*yeaGH*	Serine protein kinase (putative)	NtrC	2.5	[18,30]
**STM1303-07**	*astCABDE*	Arginine/ornithine/glutamine metabolism	NtrC	**2.4**^**f**^	[31,32]
**STM1690-86**	*pspABCDE*	Phage shock proteins	PspF	**17**	[5]
**STM2360-56**	**------***ubiX*	Amino acid transport (putative)	STM2361	**100**	[18]
**STM2840-41**	*norV ygbD*	Nitric oxide reductase	NorR	**16**	[18,33]
**STM2843-42**	*hydN hypF*	Hydrogenase maturation proteins	FhlA	**13**	[34]
**STM2853-44**	*hycABCDEFGHI***-**	Hydrogenase 3	FhlA	**26**	[35]
**STM2854-58**	*hypABCDE*	Formate-hydrogen lyase system	FhlA	**5.6**	[35]
**STM3521-18**	**-***rtcBA*	RNA repair system (putative)	RtcR	**71**	[23]
STM3568	*rpoH*	Heat shock sigma factor (σ^32^)		1.7	[36,37]
**STM3772-66**		PTS (putative)	STM3773	**39**	[18]
**STM4172**	*zraP*	Zinc resistance-associated protein	ZraR	**16**	[3,18]
**STM4173-74**	*hydHG*	Zinc resistance two-component system	ZraR	**3.7**	[3]
STM4244	*pspG*	Phage shock protein	PspF	1.4	[38]
**STM4285**	*fdhF*	Formate dehydrogenase	FhlA	**29**	[39]
**STM4535-40.s**		PTS (putative)	STM4534	**16**	[18]
***Novel σ***^***54***^**- *dependent operon *:**					
**STM2944-2937**		CRISPR-associated genes		**1.6**^**f**^	--

^a^Locus tags for genes within operons or sRNA genes are grouped by those previously shown to be σ^54^-dependent in *Salmonella*, previously predicted to be σ^54^-dependent, or identified in this study as encoded in a novel σ^54^-dependent transcript. Locus tags for operons that are not up-regulated are in **bold** type.

^**b**^Genes that have not been assigned a gene symbol are represented by a dash (−).

^**c**^Known or predicted bacterial enhancer-binding protein (bEBP) that activates the σ^54^-dependent operon.

^**d**^Signal ratio for the first gene in the operon in WT and Δ*rpoN* strains expressing DctD250 from pPHBP92. Operons with at least one gene with a signal ratio >3 and p-value <0.02 are considered up-regulated by RpoN; signal ratios above the 3-fold cut off are in **bold** type. Data for all genes in these operons can be found in Additional file1.

^**e**^References for operons shown to be σ^54^-dependent in *Salmonella* and for operons either determined to be σ^54^-dependent in other bacterial genera or predicted to be regulated by σ^54^ in *Salmonella* are listed.

^f^The first gene in the operon was <3-fold up-regulated, but other genes in the operon were >3-fold up-regulated.

### Known σ^54^-dependent operons and sRNA

If our hypothesis is correct, then in the presence of DctD250 we should observe up-regulation of operons (one or more structural genes) and sRNA genes that are known to have σ^54^-dependent promoters, even though they are normally activated by different bEBPs. Previously, four *Salmonella* operons have been experimentally shown to be regulated by σ^54^: *prpBCDE*[4], *glnHPQ*[27], *argT*[2], and *glnALG*[29]. Additionally, two sRNA genes, *glmY* and *glmZ*, have also been shown to have σ^54^-dependent promoters [28]. Table 2 summarizes the genes, functions, bEBPs, and microarray results for the known σ^54^-dependent operons and sRNA genes of *Salmonella*.

The DNA microarrays showed up-regulation of all four known σ^54^-dependent operons in *Salmonella*, *prpBCDE*, *glnHPQ*, *argT*, and *glnALG* (Table 2). The two sRNA genes with known σ^54^-dependent promoters did not appear up-regulated by σ^54^. This result was not surprising since in *S*. Typhimurium both *glmY* and *glmZ* possess σ^70^-dependent promoters that fully overlap the σ^54^-dependent promoters, such that the Eσ^70^ and Eσ^54^ compete for binding to their respective promoters [28]. Gopel et al. [28] demonstrated that the level of *glmY* transcription was similar in wild type and Δ*rpoN* cells and that transcription of *glmZ* actually increased in the *rpoN* mutant, reflecting that the σ^70^-dependent promoter for *glmZ* is stronger than the σ^70^-dependent promoter for *glmY*. The presence of a σ^70^ promoter does not necessarily preclude detection of a σ^54^-dependent promoter controlling expression of a gene or operon in these microarray assays, though; the promoter region of *glnA* has non-overlapping σ^70^- and σ^54^-dependent promoters [29], yet was up-regulated 48-fold. Taken together, these results for the known σ^54^-dependent promoters are consistent with our hypothesis that DctD250 can promiscuously and constitutively activate σ^54^-holoenzyme at a variety of σ^54^-dependent promoters.

### Confirmation of predicted σ^54^-dependent operons

There are 20 operons that we define as ‘predicted’ σ^54^-dependent operons in *Salmonella*. These predictions are based on *in silico* analyses indicating either homology to known σ^54^-dependent operons in *E*. *coli* and other enteric bacteria or promoter sequence homology along with genetic proximity to predicted bEBP genes [3,5,18,22,23,30-39]. However, σ^54^-dependent transcription of these operons has not previously been experimentally demonstrated in *Salmonella*. In the DNA microarrays, 16 of the 20 operons that have been predicted to have σ^54^-dependent promoters in *Salmonella* were up-regulated in WT+DctD250 as compared to Δ*rpoN*+DctD250 (Table 2), providing experimental evidence that these genes are, in fact, regulated by σ^54^ in *S*. Typhimurium LT2.

For these 16 up-regulated σ^54^-dependent operons there are 11 different bEBPs that either are known or predicted to activate expression from their σ^54^-dependent promoters (Table 2). Five of the up-regulated operons, STM0577-0572, STM0649.s-0653, STM2360-2356, STM3772-3766, and STM4535-4540.s, were predicted to be σ^54^-dependent based on linkage to a predicted bEBP and an upstream sequence with the essential -12 and -24 elements of a σ^54^-dependent promoter [18]. There are no orthologs in *E*. *coli* for the predicted bEBPs associated with these operons; three of these predicted bEBPs, STM0571, STM3773 and STM4534, are similar to the LevR-type EBPs found in Gram-positive bacteria [18]. In addition to the microarray evidence presented here for σ^54^ regulation of these operons, we know that STM3773 is the bEBP controlling expression of STM3772-3776 and that this operon encodes the components of a phosphotransferase system permease for D-glucosaminic acid and enzymes required for catabolism of this acid sugar [40]. These results show that DctD250 can activate expression at σ^54^-dependent promoters that are normally regulated by the LevR-type bEBPs.

Of the four predicted σ^54^-dependent operons that did not fulfill our criteria for upregulation in the microarray, at least two have additional σ^54^-independent promoters, which may have masked the effect of σ^54^ on transcription levels. The heat shock sigma factor gene *rpoH* has been shown to be under the control of additional promoters and other regulatory proteins in *E*. *coli*[36]. The conservation of this promoter region for *rpoH* in *S*. Typhimurium LT2 suggests that a similar complex regulatory scheme may be involved [37], thereby reducing the effects of the Δ*rpoN* mutation. The *yeaGH* operon, which was just below the 3-fold cutoff for up-regulation in the microarray analysis, has previously been shown to be under control of σ^S^ in *Salmonella*[41]; however, our assays utilized *S*. Typhimurium LT2, which has a defective *rpoS* gene due to a transversion mutation in the start codon [42]. The promoter-reporter assay with the *yeaGH* promoter region, described below, suggests there is a σ^54^- and σ^S^-independent promoter expressing the *yeaGH* operon in both the WT+DctD250 and Δ*rpoN*+DctD250 strains.

The frequency of alternate promoters seen for the σ^54^-dependent operons in *Salmonella* (at least 15% for the known and predicted promoters in our analyses) is not unique. Zhao et al. [13] estimate that 14% of σ^54^-dependent genes in *E*. *coli* are transcribed by σ^70^-associated RNA polymerase and suggest that expression of σ^54^–dependent genes from alternate promoters allows for differential expression under various environmental conditions.

### New potential σ^54^-dependent genes

In addition to the σ^54^-dependent expression of known or predicted genes and operons, the DNA microarray analysis revealed up-regulation of a gene, STM2938, which has not previously been reported or predicted to be σ^54^-dependent. STM2938 is the penultimate gene in a nine-gene operon that is annotated as a group of CRISPR-associated (*cas*) genes. Although none of the other genes in this operon seem to be controlled by σ^54^, further evidence is presented below that supports the presence of a σ^54^-dependent promoter within the gene upstream of STM2938. CRISPR (Clustered Regularly Interspaced Short Palindromic Repeats) and *cas* genes constitute an adaptive immune system in bacteria and archaea that protects against invading mobile DNA, such as phage and plasmids [43]. The response to phage infection, which is referred to as phage shock, is regulated by σ^54^ and the bEBP PspF in *E*. *coli*[5]; thus, it would not be surprising for essential components of the bacterial immune response in phage infection to be regulated similarly. The potential σ^54^-dependent gene STM2938 is a homologue of the *cas1* gene, which is an endonuclease that is associated with all CRISPR loci and is most likely involved in the adaptation phase of the CRISPR-*cas* immune system [44]. The regulation of this *cas1*-like gene by PspF in *Salmonella* is currently under investigation.

There were 12 additional ORFs that met the 3-fold cutoff for up-regulation by σ^54^ in the microarray assay, including genes for pilin biosynthesis (*hofB*), histidine ammonia lyase (*hutH*), bEBPs (*ygaA*, *fhlA*), propanediol utilization (*pduG*), siderophore production (*iroD*), and cell invasion (*invG*). The whole genome chromatin immunoprecipitation assays described below did not reveal σ^54^ binding sites associated with these ORFs; thus the expression of these genes may be indirectly affected by the absence of σ^54^ in the Δ*rpoN* mutant, or constitute false positives (Additional file 1).

### ChIP-chip analysis of genome-wide σ^54^ binding sites in *Salmonella*

In the characterization of the σ^54^ regulon of *Salmonella*, determination of the genomic binding sites for the Eσ^54^ allows confirmation of primary transcripts indicated by microarray analysis and recognition of potential σ^54^-regulated genes that might not have been detected due to instability of the transcripts. To assess the binding of Eσ^54^ in the *S*. Typhimurium LT2 genome, we isolated σ^54^-DNA complexes from WT and Δ*rpoN* strains that did not contain the DctD250 expression plasmid. Since bEBPs do not activate transcription by recruiting Eσ^54^ to promoter sequences [8], inclusion of DctD250 should not be necessary to detect binding of holoenzyme to promoter sequences in the ChIP-chip assay. Protein-DNA complexes containing either Eσ^54^ or σ^54^ are pulled down in the ChIP with α-σ^54^. The σ^54^ subunit is most likely to interact with the genome in the context of the RNA polymerase holoenzyme; however, σ^54^ has been shown to specifically bind in the absence of the core RNA polymerase at σ^54^-dependent promoters that have a T-tract upstream of the GC in the -12 promoter element [45]. DNA fragments from the α-σ^54^ ChIP were labeled and applied to the same complete open reading frame arrays as used in the microarray analysis.

Since the use of the ORF arrays did not allow direct mapping of the binding sites, we combined the ChIP-chip data with *in silico* analysis to determine the potential σ^54^ DNA binding sites. A Position-Specific Score Matrix (PSSM) was created using 27 known or previously predicted σ^54^-dependent promoters from *S*. Typhimurium LT2 (Additional file 2); the extent of each promoter sequence used for the PSSM (18 bp) was based on the consensus sequence for σ^54^-dependent promoters defined by Barrios et al. [9] and comparison analysis of the known *Salmonella* σ^54^-dependent promoters. This PSSM was applied with the Motif Locator program [46] to the enriched ORF sequence and 1000 bp of flanking sequence on both sides of the ORF to identify potential σ^54^ DNA binding sites. The size range of DNA fragments that were pulled down via ChIP and amplified by ligation-mediated PCR was 200–1000 bp long, as determined by agarose gel electrophoresis, suggesting that intergenic binding sites up to 1000 bp from the enriched ORF might be detected in the ChIP-chip assays.

### σ^54^ binding to promoters for known, predicted, and novel σ^54^-dependent operons

In the ChIP-chip assays with the WT and Δ*rpoN* strains, the promoter-proximal gene for all the 24 known and predicted σ^54^-dependent operons and the 2 sRNA genes (Table 2) were enriched, as defined by a stringent cut-off, i.e. signal ratio ≥3 and p-value <0.02 (Table 3). The associated promoter sequences, as determined by the *in silico* analysis, had PSSM scores ranging from 10.9 to 23.6 and were within 27 to 154 bp of the enriched ORF. In the DNA microarrays, six of the known or predicted σ^54^-dependent operons did not appear up-regulated; but in the ChIP-chip assays the promoter regions for all six operons gave signal ratios ranging from 3.2- to 39-fold greater in WT than in Δ*rpoN* cells. The detection of the σ^54^-dependent promoters for all the other known and predicted σ^54^-dependent operons supports the efficacy of our approach to mapping potential σ^54^-binding sites.

**Table 3:** **ChIP-chip signal ratios, PSSM scores, and predicted binding sites for ORFs enriched in the presence of σ**^**54**^

**Locus tag**^**a**^	**Gene name**	**Signal ratio**^**b**^	**Orientation**^**c**^	**PSSM**^**d**^	**Start**	**End**	**Sequence**
*Sites located within intergenic regions*:							
**STM0368**	*prpB*	11	+	20.7	417914	417931	TGGCATAGCCTTTGCTTT
STM0448	*clpP*	4.6	+	14.6	503028	503045	TGTCACGTATTTTGCATG
**STM0462**	*glnK*	3.2	+	20.0	520445	520462	TGGCACATCCTTTGCAAT
**STM0577**		8.3	+	17.9	636883	636866	TGGCACGCCGTTTGCCAT
**STM0649.S**		6.9	+	18.7	711945	711962	TGGCACGCCTTTTGATTA
**STM0665**	*gltI*	3.2	+	22.1	730107	730090	TGGCACGTCTATTGCTTT
**STM0830**	*glnH*	16	+	20.8	897079	897062	TGGCATGATTTTTTCATT
**STM1285**	*yeaG*	4.1	+	21.5	1363884	1363867	TGGCATGAGAGTTGCTTT
**STM1303**	*astC*	4.0	+	21.7	1382105	1382122	TGGCACGAATGCTGCAAT
**STM1690**	*pspA*	23	+	20.3	1782486	1782469	TGGCACGCAAATTGTATT
**STM2355**	*argT*	3.5	+	16.2	2466359	2466376	TGGCATAAGACCTGCATG
**STM2360**		4.8	+	23.6	2472731	2472714	TGGCATGCCTTTTGCTTT
**STM_R0152**	*glmY*	31	+	20.6	2707874	2707857	TGGCACAATTACTGCATA
STM2809	*proV*	9.5	-	14.6	2955839	2955822	TGGCATGAATATTGCGAG
**STM2840**		6.5	+	20.1	2985009	2985026	TGGCACACTAGCTGCAAT
**STM2843**	*hydN*	23	+	17.1	2990721	2990704	TGGCACGATTCGTGTATA
**STM2853**	*hycA*	31	+	17.9	2999639	2999622	TGGCATGGAAAATGCTTA
**STM2854**	*hypA*	71	+	22.4	2999753	2999770	TGGCATAAATATTGCTTT
**STM3521**	*rsr*	15	+	21.2	3684734	3684717	TGGCACGCTGGTTGCAAT
**STM3568**	*rpoH*	22	+	18.9	3736836	3736819	TGGCACGGTTGTTGCTCG
**STM3772**	*dgaA*	3.6	+	20.3	3972484	3972467	TGGCACAACCTTTGCTCT
**STM_R0167**	*glmZ*	15	+	19.5	4141620	4141637	TGGCACGTTATGTGCAAT
**STM4007**	*glnA*	4.2	+	19.2	4217110	4217093	TGGCACAGATTTCGCTTT
**STM4172**	*zraP*	29	+	17.4	4388217	4388234	TGGCACGGAAGATGCAAG
**STM4173**	*hydH*	4.8	+	20.1	4388385	4388402	TGGCATGATCTCTGCTTA
**STM4244**	*pspG*	39	+	19.4	4465042	4465059	TGGCATGATTTTTGTAAG
**STM4285**	*fdhF*	10	+	18.2	4527564	4527547	TGGCATAAAACATGCATA
STM4367	*yjeB*	3.8	+	14.1	4610407	4610424	TGGCAGATATTTTGCTTG
**STM4535**		12	+	18.3	4794881	4794898	TGGCACGCCGCTTGCTCT
*Sites located within the enriched ORF*:							
STM0131	*ftsQ*	7.6	+	6.2	153598	153615	TGGAACGCGTCTTGCAGG
STM0155		4.1	+	9.5	182767	182784	CGGCATGGCATTTGCCAG
STM0322	*proA*	7.8	-	11.3	368058	368041	CGGCACAGTTTATGCAAG
STM0332		3.0	-	8.1	376286	376269	TGGCCAGAAATATGCTTA
STM0526	*ylbA*	4.3	+	9.1	588233	588216	TGGCATTAATGCTGCATC
STM0699		14	+	13.7	761691	761674	TGGCATCGATATTGCAAA
STM0879^^^	*potH*	5.2	+	12.1	951550	951567	TGGCAGGAGTTTTTCAAT
STM0884	*ulaA*	5.2	+	10.0	955545	955562	CGGCACGATTTTTTCCAT
STM0901		3.9	+	11.7	971761	971778	TGGCATGAAACTTGTCAC
STM0940		9.5	+	13.7	1018097	1018080	TGGCCTGAATCTTGCTAA
STM0961		7.6	-	17.7	1041686	1041669	TGGCATGAAAGCTGCTCA
STM1361	*ydiM*	3.6	+	11.6	1443903	1443886	TGGCATTCTTTATGCTCA
STM1390	*orf242*	8.9	-	12.9	1475563	1475546	TGGCATCATTATTGCCTA
STM1409	*ssaJ*	5.0	+	6.5	1490273	1490290	TGGCATGAAGGTTCATCG
STM1586		6.6	-	13.5	1672845	1672862	TGGCAAGAATATTGCCAT
STM1594	*srfB*	4.8	+	13.4	1681565	1681582	TGGCACACGTTTTGCGCT
STM1665		4.2	-	11.7	1759185	1759168	TGGCATCATTTTTTCAAG
STM1904^^^	*yecN*	3.6	+	5.9	1998988	1999005	TGGCAAACCTGTGGTATA
STM1928	*otsA*	5.3	+	8.1	2023398	2023381	TGGCAGGAGCGTTTTATT
STM1990	*yedA*	5.6	+	14.4	2072998	2073015	TGGCGCGCTTTTTGCCTT
STM2033	*cbiC*	4.4	-	2.2	2111221	2111238	CGGTATAAATAATGCACG
STM2115	*wcaA*	4.3	-	9.5	2198775	2198792	TGGCATATAAATTGAGAT
STM2181	*yohJ*	15	+	4.9	2277993	2278010	AGGCATTTTTCTTGCATC
STM2430	*cysK*	4.8	-	11.7	2544207	2544190	TGGCATCACTGTTGCAGT
STM2475		9.0	-	1.0	2585621	2585638	TGGCACATCAGGCAAAAG
STM2476	*ypfG*	3.1	+	12.7	2586874	2586857	TGGCAGGTCACCTGCAAT
STM2517	*sinH*	4.6	-	11.1	2650462	2650479	TGGTACGGATCTTGCCAT
STM2563	*yfhG*	4.7	-	6.8	2705786	2705803	CGGCGTAATTTTTGCATC
STM2939	*ygcH*	10	+	10.9	3080061	3080044	CGGCACAGCTCTTGCATC
STM2957	*rumA*	5.5	+	14.5	3105809	3105792	TGGAACGCTTTTCGCATT
STM3072		4.1	-	6.9	3234181	3234164	TGGCCCATTGAATGCATC
STM3302	*yhbE*	5.5	+	12.6	3472042	3472025	TGGCATGATGGTCGCCAG
STM3535	*glgA*	8.0	+	11.6	3702315	3702298	AGGCATGTTTTATGCAAA
STM3721	*rfaP*	13.5	+	8.3	3916283	3916300	TGGTACGTAAAATGCACG
STM3863^^^		7.5	+	11.1	4072959	4072942	TGGCGCGATTATTGCCAG
STM3919	*wzzE*	4.2	+	11.0	4128295	4128312	TGGCCTGCTATTTGCCCT
STM3924	*wecD*	22	+	11.8	4133232	4133249	TGGCGCGGAAATTGCACA
STM4013.S		3.6	-	13.6	4222708	4222725	TGGCATAAAACCTGAAAA
STM4226	*yjbA*	6.4	-	4.2	4446318	4446301	AGGCGCGAATAATGCATC
STM4290	*proP*	13	+	10.1	4532022	4532039	TGGCCTGATTTTTGCAGG
STM4572	*stjB*	8.3	-	8.2	4826908	4826925	TGGCGTGGCGATTTCAAT

^a^Loci listed in **bold** are known or predicted σ^54^-dependent promoters.

^b^Ratio of signals between WT and Δ*rpoN* cells.

^c^Orientation of the predicted binding site with respect to the listed ORF. (+) binding site is in *same* direction as ORF; (−) binding site is in *opposite* direction as ORF.

^d^PSSM score for the best predicted binding site within 1 kb of enriched ORF using the position-specific scoring matrix derived from the sequences in Additional file 2. As a reference to interpret the PSSM scores, the *S*. Typhimurium LT2 chromosome contains 3 sites with PSSM scores ≥22.0, 21 sites with PSSM scores ≥18.0, 61 sites with PSSM scores ≥14.0, and 401 sites with PSSM scores ≥10.0.

^The predicted binding site is a potential promoter for a neighboring gene based on its orientation and location within 250 bp of the 5’ end of a neighboring gene or a long intergenic region (>100 bp) that may encode a sRNA.

The ChIP-chip analyses also showed that only one (STM2938) of the 13 newly-identified, potential σ^54^-dependent operons from the DNA microarray assays has a σ^54^ DNA binding site associated with it (Table 3), suggesting that the other 12 operons may be indirectly regulated by σ^54^. The σ^54^ DNA binding site associated with STM2938, the *cas1*-like gene, is within the upstream gene, STM2939 (539 bp from the start of STM2938). Further characterization of this potential σ^54^ promoter is described below in the promoter-reporter analysis.

### σ^54^ binding to newly identified potential promoter and regulatory sites

**Figure 2:**
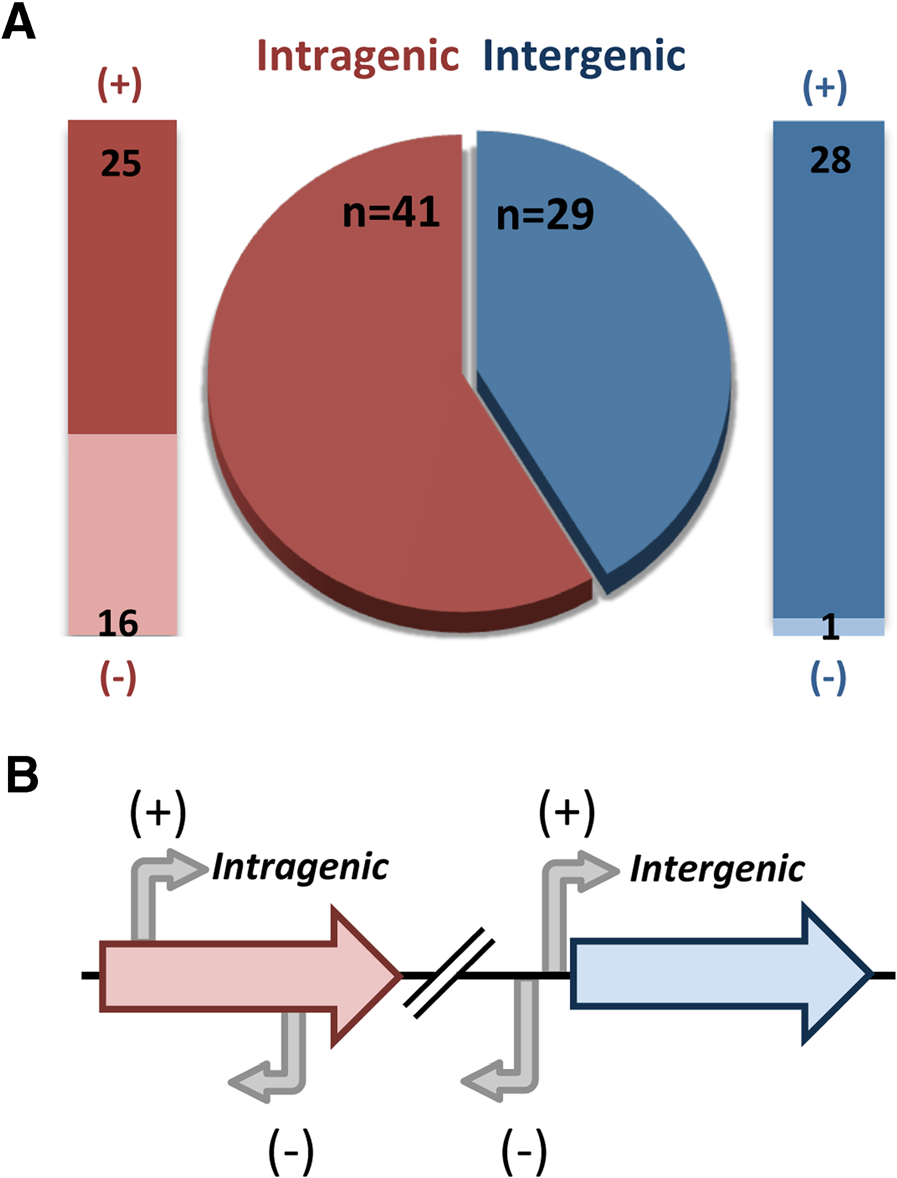
**Binding sites predicted by ChIP-chip analysis. A)** Location of the predicted binding sites for the 70 ORFs enriched by α-σ^54^ pulldown. Outer bars represent further breakdown by location and orientation of the binding site relative to the enriched ORF, as diagrammed in **(B)**. A (+) indicates that the binding site is in the same orientation as the ORF while (−) indicates that the binding site is in the opposite orientation as the enriched ORF.

In total, 70 ORFs were each found to be enriched in 3 replicate samples for the WT cells as compared to the Δ*rpoN* cells in the ChIP-chip assays (Table 3). The potential σ^54^ binding site with the highest PSSM score within each enriched ORF or up to 1000 bp of flanking intergenic sequence was identified by Motif Locator and is reported in Table 3. For the 70 enriched ORFs, 29 of the associated binding sites mapped to intergenic regions and 41 of the potential σ^54^ binding sites were located within the enriched ORF (Figure 2). In determining the most likely binding site for an enriched ORF, sequence within an adjacent non-enriched ORF was not considered for potential σ^54^ binding sites, since the ORF containing the binding site should be enriched; therefore, even if a site with a higher PSSM score was located in an immediately adjacent non-enriched ORF, the next highest scoring site found in either the enriched ORF or adjacent intergenic sequence was reported as the potential binding site in Table 3. This reflects a limitation of the *in silico* prediction of σ^54^ binding sites based on a PSSM that was created with known and predicted intergenic promoter sequences; the sequences for intragenic promoters or for σ^54^ binding sites that are regulatory sites, but not promoters, may differ enough to appreciably affect PSSM scores.

**Figure 3:**
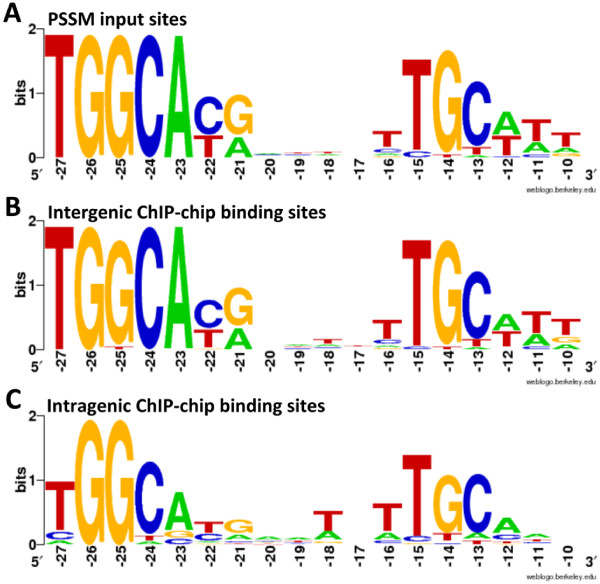
**Alignment of σ**^**54 **^**binding sites.** Weblogos show the consensus sequence for **A)** 27 known/predicted promoter sequences used to generate the position-specific scoring matrix **B)** 29 predicted intergenic binding sites for ORFs enriched in ChIP-chip analysis or **C)** 41 predicted intragenic binding sites from within ORFs enriched in ChIP-chip analysis. Weblogos were generated using the online program available at http://weblogo.berkeley.edu/.

Consensus sequences were generated using WebLogo [47] for the intergenic and intragenic potential σ^54^ binding sequences and for the promoter sequences used to generate the PSSM (Figure 3). Noteworthy differences in the consensus sequence for the intragenic σ^54^ binding sites, as compared to the consensus sequences for the intergenic σ^54^ binding sites and PSSM promoters, are at the −23 and −11 positions, which each contribute in different ways to σ^54^-promoter DNA interactions. The −23 A-T base pair is important in promoter recognition by σ^54^; the winged helix-turn-helix DNA binding motif of σ^54^ makes base-specific contacts with the top strand GG at positions −26 and −25 and with the bottom strand T at position −23 [48]. The base pairs immediately adjacent to the conserved GC element in the -12 region of the promoter are involved in Eσ^54^ binding to form the stable closed complex; the bases on the bottom strand of the promoter at the -12 and -11 positions interact with σ^54^ in a short region of ‘early melting’ that stabilizes closed complex until the bEBP binds σ^54^ and activates the holoenzyme to transition to open complex [49]. The reduced conservation of nucleotide sequence at the −23 and −11 positions for the potential intragenic σ^54^ binding sites may reflect varied functionality of these intragenic sites, or a level of inaccuracy inherent to *in silico* prediction of the binding sites associated with enriched ORFs in the ChIP-chip assays.

The position and orientation for each potential σ^54^ binding site are indicated in Table 3 and summarized for all the binding sites in Figure 2. This information is useful in considering possible functions for the binding sites. For example, the 16 intragenic σ^54^ binding sites oriented in the opposing direction of the gene might regulate by transcription interference and/or anti-sense RNA [19]. Four intragenic σ^54^ binding sites are within 250 bp of the 5’ end of a downstream gene, or a large intergenic region (>100 bp), and oriented in the direction such that they might act as promoters for the downstream gene or a sRNA [20]. Binding sites located near a functional σ^54^ promoter may serve to accelerate the search for the promoter by Eσ^54^ sliding from the secondary sites [50]; while binding sites adjacent to, or overlapping, a σ^70^- or σ^54^-promoter may bind Eσ^54^ or σ^54^ and repress or activate transcription from the other promoter [51]. The possible functions of the σ^54^ binding sites are quite varied and many are dependent on whether the binding site can function as a promoter.

It is likely that our initial approach to defining the global binding sites of σ^54^ in *S*. Typhimurium LT2 resulted in an underestimation of the number of binding sites. Multiple sites within ~2,000 bp of an ORF would enrich one or two adjacent ORFs and, in our analysis, would have been counted as one site. In addition, since Eσ^54^-promoter closed complexes are reversible [52], some complexes might have not been detected due to high disassociation rates; detection of these sites may be improved in the presence of DctD250, which stimulates conversion of closed complex to the more stable open complex, and rifampicin, which prevents extension of RNA past the second or third nucleotide [53], thus improving the chances of cross-linking Eσ^54^ at the promoter sequence [54].

### Promoter-reporter analysis to determine activity for predicted promoter sequences

To assess the functionality of σ^54^ binding sites defined by the ChIP-chip and PSSM analyses, promoter-*lacZ* fusion assays were performed using several of these sequences. We had two goals in performing these assays. First, we wanted to further confirm the σ^54^-dependent promoter activity for some of the predicted σ^54^-dependent promoters that were up-regulated in the DNA microarrays and enriched in the ChIP-chip assays. Secondly, we wanted to test the σ^54^ binding site predictions from the ChIP-chip combined with PSSM analyses; i.e. does a potential σ^54^ binding site equate to a σ^54^-dependent promoter? This promoter function assay is the initial exploration of the roles for σ^54^ binding sites located within intragenic regions.

**Figure 4:**
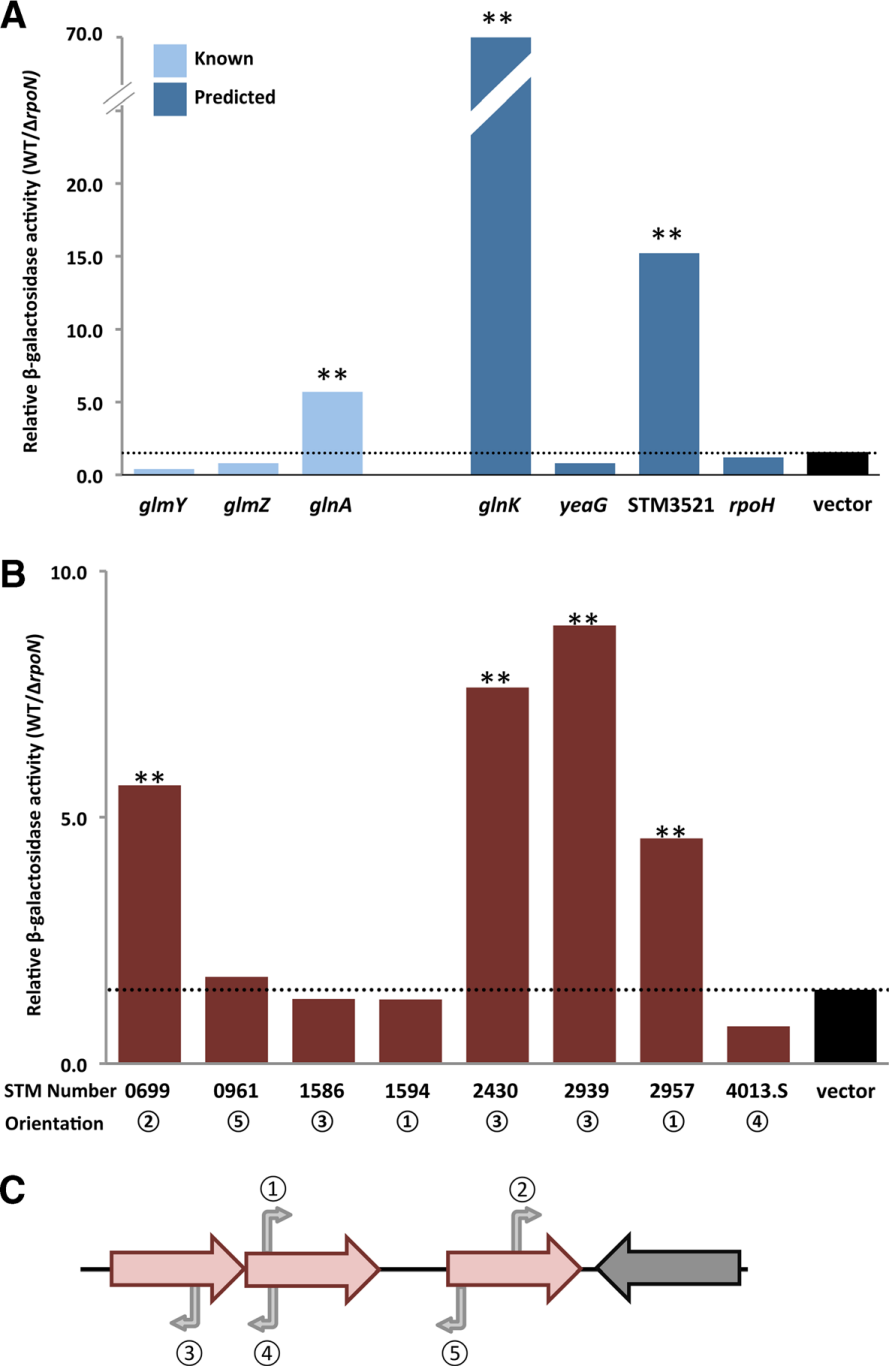
**Promoter location, orientation, and activity for selected σ**^**54 **^**binding sites.** The ratio of β-galactosidase activity (Miller Units) in WT+DctD250 vs. Δ*rpoN*+DctD250 cells is shown for **(A)** known (light blue bars) and predicted (dark blue bars) σ^54^-dependent promoters, and **(B)** potential intragenic promoter sequences (red bars) in the promoter reporter vectors, pDS11 or pDS12 (black bars). Double asterisks denote significant increase in β-galactosidase activity in WT+DctD250 versus Δ*rpoN*+DctD250 (p-value <0.02). Circled numbers below locus tags indicate orientation of the potential promoter sequence, as illustrated in **(C)**. Orientation of potential intragenic promoter sequence is: **1)** same as ORF and >300 bp from 3’ end; **2)** same as ORF and <300 bp from 3’ end of a convergent downstream gene; **3)** opposite of ORF and >300 bp from 5’ end; **4)** opposite of ORF and <300 bp from the 3’ end of an upstream gene; and **5)** opposite of ORF and <300 bp from 5’ end of gene, but >300 bp from the 3’ end of an upstream gene.

Potential promoters were introduced upstream of a promoter-less *lacZ* gene in a reporter vector, either pDS11 or pDS12 (which differ only in their MCS sequence). These promoter-reporter plasmids were co-transformed along with the DctD250 expression plasmid into either WT or Δr*poN* cells. After induction of DctD250 expression, standard β-galactosidase assays were performed. The results from WT were compared to those from the Δ*rpoN* mutant to determine whether activity seen was σ^54^-dependent (Figure 4). For intergenic sequences that were known or predicted to be σ^54^-dependent promoters, the results matched those observed in the DNA microarray assays (Figure 4A, Table 2). The *glnA*, *glnK*, and STM3521 (*rtcBA* operon) promoters showed strong σ^54^-dependent activity. For the *glmY*, *glmZ*, *rpoH*, and *yeaG* promoters, transcription in the Δ*rpoN* mutant was either as high as or higher than in wild type cells. This is likely due to the presence of σ^70^-type promoters in the cloned sequence. In addition to the σ^54^ dependent promoters, other promoters have been reported upstream of *glmY*, *glmZ*, *rpoH*, and *yeaG*[28,36,41].

A total of eight intragenic sites identified in the ChIP-chip assay were selected for functional analysis (Figure 4B). All of these predicted sites had PSSM scores >10. As shown in Figure 4C, the sites chosen represent a variety of configurations with regard to their position and orientation within the ORF as well as the position and orientation of downstream ORFs. Given the possible functions for an intragenic promoter sequence (e.g. promoter for a downstream gene or sRNA, generation of antisense RNA, etc.), results of our analysis allow us to determine which, if any, of these roles may be attributable to any of these promoters.

Comparing the levels of *lacZ* expression in wild type cells to those in Δ*rpoN* mutants, we found that four of the eight intragenic sites were able to function as σ^54^-dependent promoters. For these sites, the difference observed between WT and Δ*rpoN* cells varied from 4.6-fold for the sequence located in STM2957 to 8.9-fold for the sequence within STM2939. Overall, the level of transcription from these promoters was relatively low, with Miller units ranging from ~30-100. The low activity levels may indicate that Eσ^54^ has a low affinity for these sequences or that the DctD250 is inefficient in productively engaging closed complexes formed at these sites. A subset of the promoter-reporter plasmids with intragenic sites that exhibited σ^54^-dependent transcription were also assayed in WT cells versus WT-DctD250 to determine the dependence of transcription on the promiscuous, constitutive bEBP (Table 1). All three intragenic promoters assayed, STM0699, STM2430, and STM2939, gave low levels of β-galactosidase activity in the absence of DctD250 and from 4.7- to 34-fold higher levels of β-galactosidase activity in the presence of DctD250. The σ^54^-dependent transcription from intragenic binding sites suggests previously unrecognized regulatory functions for σ^54^ in *Salmonella*; however, it will be critical to characterize transcription from their chromosomal loci before biological functions can be ascribed.

Some of the potential promoter sequences that were assayed failed to show any transcriptional activity. There are a number of possible reasons for the lack of promoter activity for these sites. Two likely explanations are: 1) the wrong sequence was chosen as the binding site based on the PSSM score and proximity to the enriched ORF in the ChIP-chip assays, i.e. a lower scoring sequence near the enriched ORF was the actual σ^54^ binding site; or 2) the σ^54^ binding site does not function as a promoter but serves another regulatory role, such as an operator site for regulating promoter activity, a site for transient binding in facilitated diffusion, or a site for sequestering Eσ^54^ in order to increase local concentration (since σ^70^ has a higher affinity for RNAP [55]).

### Summary of *S*. Typhimurium LT2 σ^54^ regulon and comparison to σ^54^ regulons of other bacteria

**Figure 5:**
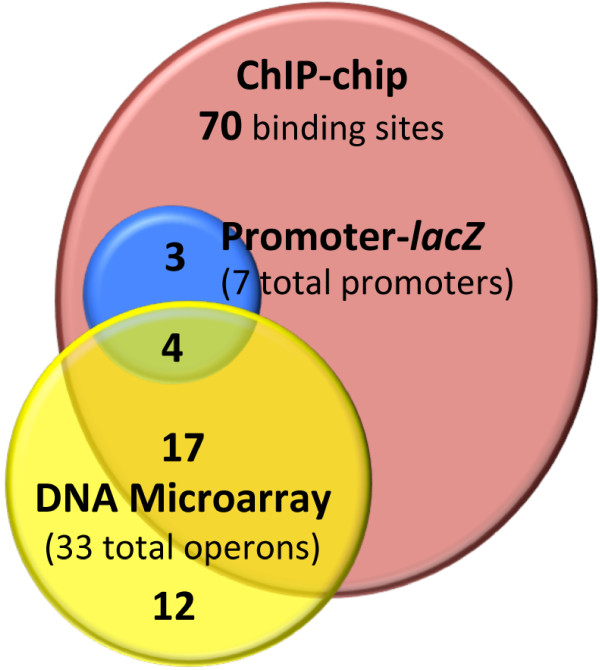
**Comparison of positive results from characterization of the σ**^**54 **^**regulon for *S*. Typhimurium LT2.** + Positive results are promoter sequences that were up-regulated >3-fold, or displayed a significant increase in β-galactosidase activity in WT+DctD250 compared to Δ*rpoN*+DctD250 in DNA microarray, and promoter-*lacZ* fusion assays, respectively or enriched >3-fold in WT compared to Δ*rpoN* cells in ChIP-chip assays. Regions of overlap indicate promoters that were positive in multiple experiments.

Figure 5 summarizes the results from the DNA microarray and promoter-fusion assays performed in the presence of DctD250 and ChIP-chip in the absence of DctD250 to characterize the σ^54^ regulon of *S*. Typhimurium LT2. Based on DNA microarray, there are 33 up-regulated operons (76 genes; Additional file 1); global ChIP-chip combined with *in silico* analysis revealed at least 70 σ^54^ binding sites (Table 3), of which 21 were associated with up-regulated operons from the DNA microarrays. The promoter-*lacZ* fusions with seven of the 29 intergenic σ^54^ binding sites and eight of the 41 intragenic σ^54^ binding sites showed DctD250- and σ^54^-dependent expression for three intergenic sites (associated with up-regulated operons) and four intragenic σ^54^ binding sites (Table 1, Figure 4). The cellular functions impacted by genes in the σ^54^ regulon of *S*. Typhimurium LT2 are quite diverse, ranging from carbon-source and amino acid metabolism to response to stressors, such as nitric oxide and toxic levels of zinc (Table 2). Our results suggest that a new cellular process may be added to this extensive list—cell immunity through the CRISPR system; the role of σ^54^ in regulating a *cas1*-related gene within an operon of CRISPR-associated genes is presently being investigated.

The σ^54^ global regulon of *S*. Typhimurium LT2 may differ from that of virulent *S*. Typhimurium isolates due to accumulated mutations in this extensively-used, laboratory strain, particularly the *rpoS* mutation that contributes to attenuation of the LT2 strain [42]. Changes in the level of expression of one sigma factor can alter the expression of genes that are expressed by different sigma factors [56]; for example, it has been shown that deletion of *rpoN* alters expression of σ^S^-dependent promoters in *E*. *coli*[57]. We are currently characterizing the σ^54^ global regulon of the virulent strain *S*. Typhimurium 14028s.

The σ^54^ regulons in other δ/γ-proteobacteria have been characterized experimentally to varying extents [13-15,58-60]. Only in *Vibrio cholera* 037 strain V52 have both global transcripts and binding sites been characterized experimentally [14]. In *E*. *coli* MG1655 and *Geobacter sulfurreducens*, the global σ^54^ transcriptomes were determined and local σ^54^ binding sites associated with up-regulated genes were assessed by computational analysis and selected promoters were assessed experimentally [13,15]. The number and diversity of the operons that are directly controlled by σ^54^-promoters in these δ/γ-proteobacteria are comparable to that of *S*. Typhimurim LT2. The greatest variability in the σ^54^ regulons of the γ-proteobacteria appears to be the location of σ^54^ binding sites. Zhao et al. [13] estimated 70 σ^54^ promoters in *E*. *coli* MG1655, of which 13 (18%) were intragenic or located between convergently transcribed genes. In *V*. *cholera*, Dong and Mekalanos [14] identified a total 68 σ^54^ binding sites, of which 35 (51%) were intragenic and, similarly, we found 70 potential σ^54^ binding sites of which 41 (58%) appear to be located in intragenic regions.

Does the success with DctD250 in characterizing the *S*. Typhimurium σ^54^ regulon predict utility of this constitutive, promiscuous activator in defining σ^54^ global regulons in bacteria from other classes in the Proteobacteria phylum, or from other phyla? The key to activation of Eσ^54^ by DctD250 in diverse bacteria is the ability of the activator to make the appropriate interactions with σ^54^ in the context of the Eσ^54^-promoter closed complex; thus, comparison of interacting regions of σ^54^ and bEBPs between *S*. Typhimurium and phylogenetically diverse bacteria is a good predictor of success. Extensive characterization of bEBP activation of Eσ^54^ in closed complex has shown that the GAFTGA motif of the AAA+ ATPase domain plays a primary and essential role for productive interactions with Eσ^54^, which lead to transcriptional activation (reviewed in [8]); the GAFTGA motif is very highly conserved among bEBPs in all bacteria that encode σ^54^, which includes bacteria from a majority of the eubacterial phyla [61]. It has not yet been determined which specific residues of σ^54^ are contacted by Loop 1 of the bEBP AAA+ ATPase domain, but it has been clearly demonstrated that multiple residues within the amino-terminal 50 amino acids of σ^54^ (Region I) are key determinants for activator interaction [62] and there is extensive conservation of amino acid sequence in Region I for σ^54^ from phylogenetically diverse bacteria [63]. Thus, the comparison of interacting regions of σ^54^ and the AAA+ ATPase domain among diverse bacteria predicts that DctD250 will be a valuable tool in characterizing the σ^54^ regulons in many bacteria.

## Conclusions

The results of DNA microarray and promoter-*lacZ* fusion analyses of the σ^54^ regulon of S. Typhimurium LT2 in the presence of DctD250 support our initial hypothesis: the AAA+ ATPase activation domain of DctD can stimulate transcription from σ^54^-dependent promoters in a constitutive and promiscuous manner, thereby facilitating the global characterization of σ^54^ regulons. Sixteen previously predicted σ^54^-dependent operons were confirmed, and a new σ^54^-dependent gene, *cas1*, was identified by the DNA microarray and ChIP-chip analyses. In addition, the ChIP-chip analyses indicate an excess of σ^54^ binding sites compared to the number of σ^54^-dependent transcripts and a high percentage of intragenic binding sites, suggesting that Eσ^54^ and σ^54^ may have more regulatory functions than transcription initiation at the start of an operon or sRNA. The number of functional promoters located inside genes suggests a need to consider such promoters in bioinformatic analyses of transcription factor binding sites.

## Methods

### Bacterial strains, media, and enzymes

The parental strain, designated wild-type, in these experiments was *Salmonella enterica subspecies enterica serovar* Typhimurium LT2 derivative MS1868 [*leuA414*(Am) *hsdSB*(r^-^m^+^)Fels^-^] [64]. An isogenic derivative, TRH134, has a deletion in *rpoN* (*ntrA*) from codons 8 through 455, rendering it auxotrophic for glutamine [65]. *S*. Typhimurium strains were cultured in either nutrient broth (NB; Difco Laboratories), MOPS minimal media [66], or nitrogen-limiting MOPS [67]. Media supplement concentrations were 5 mM L-glutamine (Gln), 40 μg/ml L-Leucine (Leu), and 10 mM L-glutamate (Glu). Cloning procedures were performed in *E*. *coli* DH5α cultured in Luria-Bertani medium (LB; Fisher Scientific). All strains were grown at 37°C. Antibiotics (Sigma-Aldrich) were used at the following concentrations (μg/ml) for *E*. *coli*/*S*. Typhimurium (NB)/ *S*. Typhimurium (MOPS), respectively: ampicillin (Amp) 80/120/50; spectinomycin (Spc) 50/125/50; streptomycin (Str) 25/75/0. All enzymes were purchased from New England Biolabs, unless otherwise indicated, and were used according to manufacturer’s recommendations.

### Plasmids

Plasmid pPBHP92 is a derivative of the expression vector pTrcHisC (Invitrogen) that expresses the *Sinorhizbium meliloti* DctD AAA+ ATPase domain (E141-S390, designated DctD250) with an N-terminal 6x-His tag. This plasmid was constructed by digestion of pHX182 [17] with NdeI, filling in the 5’-overhang with T4 DNA polymerase and subsequent digestion with XhoI. The blunt-XhoI fragment containing the truncated *dctD* was cloned into pTrcHisC, which had been cut with NheI, blunt-ended, and cut with XhoI. The truncated *dctD* is under control of P_trc_ and subject to repression by the vector-encoded LacI. The reporter plasmids used in these studies, pDS11 and pDS12, are both derivatives of pDV6 [25] that contain a promoter-less copy of *lacZ* downstream of a MCS region. The MCS region was generated by annealing two oligonucleotide primers (Additional file 3) which were then ligated into a pDV6 backbone that had been digested with BamHI and HindIII. pDS11 and pDS12 differ only in MCS sequence. Potential promoter sequences were amplified from *S*. Typhimurium LT2 genomic DNA using Taq polymerase and the primers in (Additional file 3) and cloned into pCR2.1 (Invitrogen). Sequencing analysis to determine accuracy and orientation was performed for all plasmids by Genewiz, Inc. (South Plainfield, NJ). Depending on their orientation in pCR2.1 potential promoter sequences were sub-cloned into pDS11/12 using XbaI and either KpnI or HindIII. Plasmid pTG4, which encodes the DctD AAA+ ATPase domain under control of P_tac_/*lacI*^*q*^, was created by amplifying the corresponding region of pPBHP92 using primers DctD-F/R (Additional file 3), digesting the product with BamHI and HindIII, followed by ligation into the similarly digested pKH66 [68].

### Transcriptional profiling by microarrays

*S*. Typhimurium strains MS1868 and TRH134, each bearing plasmid pPBHP92 (WT+DctD250 and Δ*rpoN*+DctD250, respectively), were grown overnight at 37°C in NB-Amp. Cultures were sub-cultured in fresh medium and grown to mid-log phase (OD_600_ ≈ 0.8). Since the basal level of DctD250 expression from pPBHP92 was shown to optimally activate transcription from a σ^54^-dependent *dctA*’-‘*lacZ* reporter [17], IPTG induction was not used for these cultures. RNA isolated using the RNAeasy kit (Qiagen) was used to generate differentially labeled cDNA using reverse transcriptase as previously described [69]. Labeled cDNA was hybridized to DNA microarrays containing complete open reading frames (ORFs) from *S*. Typhimurium LT2 printed in triplicate [70]. Microarrays were scanned with a ScanArray Lite laser scanner (Packard BioChip Technologies, Billerica, MA) using ScanArray Express 1.1 software. Signal intensities were quantified using QuantArray 3.0 (Packard). The ratio of WT+DctD250 signal to Δ*rpoN*+DctD250 signal was determined for each of the triplicate spots and the median value for each ORF was used in the statistical analysis [70]. Data shown is the result of three biological replicates with statistical analysis performed using the WebArrayDB program [71,72]. The intensity values for the three biological replicates of WT+DctD250 and of Δ*rpoN*+DctD250 were compared for the calculation of the p-values, where the null hypothesis was that the intensities for WT+DctD250 and Δ*rpoN*+DctD250 would be equivalent. Genes that displayed a WT+DctD250/Δ*rpoN*+DctD250 signal ratio of >3-fold with a p-value of <0.02 were considered to be up-regulated.

### Chromatin immunoprecipitation (ChIP)

ChIP was carried out using the ChIP Assay kit (USB Corporation) essentially as described by the manufacturer’s instructions. Briefly, 100 ml cultures of *S*. Typhimurium strains MS1868 (WT) and TRH134 (Δ*rpoN*) were grown overnight in NB at 37°C and sub-cultured in fresh medium the next day. Once cultures reached mid-log phase (OD_600_ ≈ 0.7), cells were treated with formaldehyde (3 ml of a 37% solution per 100 ml of culture) for 10 min. at room temperature to cross-link proteins to DNA. Cross-linking was quenched by the addition of glycine (10 ml of 1.33 M solution per 100 ml of culture) and incubation at 4°C for 30 min. Cells were harvested, washed and lysed in accordance with kit instructions. Cells were lysed in two passages through a French pressure cell at 10,000 psi. Cell extracts were clarified and pre-cleared with the provided protein A-Sepharose bead slurry per the kit instructions. 0.6 ml of the resulting extracts were mixed with 2 μl of rabbit anti-serum against *S*. Typhimurium σ^54^[73] and incubated with gentle shaking overnight at 4°C. The next day, 50 μl of protein A-Sepharose bead slurry was added to each sample, incubated 1 hr at room temperature and collected by centrifugation. The beads were washed, and protein-DNA complexes were eluted from the beads and disrupted per the supplier’s instructions. DNA was purified from each sample using the Qiagen PCR purification kit.

### ChIP-chip assays

Purified ChIP DNA was amplified by ligation-mediated PCR, adapting the procedure found at [http://www.flychip.org.uk/protocols/archive_protocols/lm_pcr.php]. Linkers consisting of complementary oligonucleotides (LM-PCR; Additional file 3) were ligated to the ends of purified DNA repaired with T4 DNA polymerase. Ligated was purified using the Qiagen PCR purification kit and the DNA was amplified with Taq polymerase (Fermentas; Burlington, ON) using LM-PCR-R as the PCR primer and the following cycling conditions: 55°C—2 min (1×); 72°C—5 min (1×); 94°C—5 min (1×); 94°C—1 min, 55°C—1 min, 72°C—1 min (24×); 72°C—5 min (1×); 4°C—hold. The resulting amplicons, most of which were 300–800 bp, were purified using the Qiagen PCR purification kit and to prepare dye-labeled DNA (Cy3 or Cy5) for hybridization to the *S*. Typhimurium complete ORF microarray. Microarrays were scanned and analyzed as above. ChIP-chip was performed on three biological replicates for the WT and Δ*rpoN* strains; the statistical analysis of the data was performed as described for the microarray data.

### Identifying candidate σ^54^ binding sites in the *S*. Typhimurium genome

The Motif Locator program [http://www.cmbl.uga.edu/software.html] was used to identify candidate σ^54^ binding sites. The program applies the standard position-specific score matrix (PSSM) described in [46]. We used a PSSM derived from the alignment of 27 high-confidence sites supported by experimental evidence in either *Salmonella* or *E*. *coli* (Additional file 2). Background nucleotide frequencies were assigned in accordance with the genomic G+C content. Pseudo-counts equal to the background frequencies were used in PSSM construction. For ORFs discovered in the ChIP-chip assay, this matrix was used to determine the most likely binding site either within the ORF itself or in the region ±200, 500, or 1000 bp surrounding the gene.

### β-Galactosidase assays

The DctD250 expression plasmid pTG4 was introduced into *S*. Typhimurium MS1868 and TRH134 by electroporation using a GenePulser 2 system (BioRad; Hercules, CA) and the resulting transformants were electroporated with pDS11 or pDS12 reporter constructs containing potential σ^54^-dependent promoter sequences. Overnight cultures grown in MOPS-LeuGln, or nitrogen-limiting MOPS-Glu, with the appropriate antibiotics were sub-cultured into fresh medium, grown to OD_600_ ≈ 0.2, and induced with 50 μM IPTG (empirically determined IPTG concentration for optimal expression of DctD250 from pTG4 to activate known σ^54^-dependent promoters on the reporter plasmids). Cultures were induced for 6 hours and β-galactosidase activity was measured as described previously [74] with the following changes: 1) assays were performed at 37°C, and 2) after stopping reaction, samples were centrifuged and OD_420_ of the supernatant was measured, eliminating the OD_550_ correction for cell debris. Activity was calculated as Miller units: [(OD_420_ × 1000)/(OD_600_ × Time(min) × volume (ml))] [74]. Ratios of activity in wild type/Δ*rpoN* cells were compared and analyzed using a 2-tailed Student’s T-test. Data shown for each promoter construct represents ≥3 biological replicates.

### Accession number for microarray and ChIP-chip data

The DNA microarray and ChIP-chip data were deposited in NCBI GEO under accession number GSE25849.

## Abbreviations

Amp: Ampicillin; bEBP: Bacterial enhancer-binding protein; ChIP: Chromatin immunoprecipitation; Eσ: RNA polymerase holoenzyme; LB: Luria-bertani media; LM-PCR: Ligation-mediated PCR; NB: Nutrient broth; RNAP: RNA polymerase; Spc: Spectinomycin; Str: Streptomycin; UAS: Upstream activation sequence.

## Competing interests

The authors declare that they have no competing interests.

## Authors’ contributions

DS harvested RNA, carried out promoter-reporter assays, and drafted and revised and prepared the manuscript. JF prepared cDNA and preformed DNA microarrays assays and applied DNA from the ChIP to the microarrays. SP and MM produced the microarrays and protocols and performed the statistical analysis of the microarray therein. JM performed the bioinformatic analyses. TH conceived, designed, and coordinated the study, harvested RNA, and performed ChIP pulldowns. AK conceived, designed, and coordinated the study, harvested RNA, and drafted and revised the manuscript. All authors read and approved the final manuscript.

## Supplementary Material

Additional file 1 **Complete list of microarray results.** The file contains data for *all* genes within operons that showed up-regulation by σ^54^. Click here for file

Additional file 2 Sequences used to generate Position-Specific Scoring Matrix. Click here for file

Additional file 3 Oligonucleotides used. Click here for file

## References

[B1] Gruber TM Gross CA Multiple sigma subunits and the partitioning of bacterial transcription space Annu Rev Microbiol 2003 57 441 466 10.1146/annurev.micro.57.030502.090913 14527287

[B2] Ferro-Luzzi Ames G Nikaido K Nitrogen regulation in *Salmonella typhimurium*. Identification of an NtrC protein-binding site and definition of a consensus binding sequence EMBO J 1985 4 2 539 547 2862031 10.1002/j.1460-2075.1985.tb03662.x PMC554219

[B3] Leonhartsberger S Huber A Lottspeich F Bock A The *hydH*/*G* Genes from *Escherichia coli* code for a zinc and lead responsive two-component regulatory system J Mol Biol 2001 307 1 93 105 10.1006/jmbi.2000.4451 11243806

[B4] Palacios S Escalante-Semerena JC *prpR*, *ntrA*, and *ihf* functions are required for expression of the *prpBCDE* operon, encoding enzymes that catabolize propionate in *Salmonella enterica* serovar Typhimurium LT2 J Bacteriol 2000 182 4 905 910 10.1128/JB.182.4.905-910.2000 10648513 PMC94363

[B5] Weiner L Brissette JL Model P Stress-induced expression of the *Escherichia coli* phage shock protein operon is dependent on sigma^54^ and modulated by positive and negative feedback mechanisms Genes Dev 1991 5 10 1912 1923 10.1101/gad.5.10.1912 1717346

[B6] Niehus E Gressmann H Ye F Schlapbach R Dehio M Dehio C Stack A Meyer TF Suerbaum S Josenhans C Genome-wide analysis of transcriptional hierarchy and feedback regulation in the flagellar system of *Helicobacter pylori* Mol Microbiol 2004 52 4 947 961 10.1111/j.1365-2958.2004.04006.x 15130117

[B7] Wigneshweraraj S Bose D Burrows PC Joly N Schumacher J Rappas M Pape T Zhang X Stockley P Severinov K Modus operandi of the bacterial RNA polymerase containing the σ^54^ promoter-specificity factor Mol Microbiol 2008 68 3 538 546 10.1111/j.1365-2958.2008.06181.x 18331472

[B8] Bush M Dixon R The role of bacterial enhancer binding proteins as specialized activators of sigma^54^-dependent transcription Microbiol Mol Biol Rev 2012 76 3 497 529 10.1128/MMBR.00006-12 22933558 PMC3429621

[B9] Barrios H Valderrama B Morett E Compilation and analysis of sigma(54)-dependent promoter sequences Nucleic Acids Res 1999 27 22 4305 4313 10.1093/nar/27.22.4305 10536136 PMC148710

[B10] Ghosh T Bose D Zhang X Mechanisms for activating bacterial RNA polymerase FEMS Microbiol Rev 2010 34 5 611 627 20629756 10.1111/j.1574-6976.2010.00239.x

[B11] Belitsky BR Sonenshein AL An enhancer element located downstream of the major glutamate dehydrogenase gene of *Bacillus subtilis* Proc Natl Acad Sci USA 1999 96 18 10290 10295 10.1073/pnas.96.18.10290 10468601 PMC17881

[B12] Reitzer LJ Magasanik B Transcription of *glnA* in *E*. *coli* is stimulated by activator bound to sites far from the promoter Cell 1986 45 6 785 792 10.1016/0092-8674(86)90553-2 2871943

[B13] Zhao K Liu M Burgess RR Promoter and regulon analysis of nitrogen assimilation factor, sigma^54^, reveal alternative strategy for *E*. *coli* MG1655 flagellar biosynthesis Nucleic Acids Res 2010 38 4 1273 1283 10.1093/nar/gkp1123 19969540 PMC2831329

[B14] Dong TG Mekalanos JJ Characterization of the RpoN regulon reveals differential regulation of T6SS and new flagellar operons in *Vibrio cholerae* O37 strain V52 Nucleic Acids Res 2012 40 16 7766 7775 10.1093/nar/gks567 22723378 PMC3439928

[B15] Leang C Krushkal J Ueki T Puljic M Sun J Juarez K Nunez C Reguera G DiDonato R Postier B Genome-wide analysis of the RpoN regulon in *Geobacter sulfurreducens* BMC Genomics 2009 10 331 10.1186/1471-2164-10-331 19624843 PMC2725144

[B16] Brahmachary P Dashti MG Olson JW Hoover TR *Helicobacter pylori* FlgR is an enhancer-independent activator of σ^54^-RNA polymerase holoenzyme J Bacteriol 2004 186 14 4535 4542 10.1128/JB.186.14.4535-4542.2004 15231786 PMC438555

[B17] Xu H Gu B Nixon BT Hoover TR Purification and characterization of the AAA+ domain of *Sinorhizobium meliloti* DctD, a sigma^54^-dependent transcriptional activator J Bacteriol 2004 186 11 3499 3507 10.1128/JB.186.11.3499-3507.2004 15150237 PMC415754

[B18] Studholme DJ Enhancer-dependent transcription in *Salmonella enterica* Typhimurium: new members of the sigma^N^ regulon inferred from protein sequence homology and predicted promoter sites J Mol Microbiol Biotechnol 2002 4 4 367 374 12125817

[B19] Georg J Hess WR cis-antisense RNA, another level of gene regulation in bacteria Microbiol Mol Biol Rev 2011 75 2 286 300 10.1128/MMBR.00032-10 21646430 PMC3122628

[B20] Gottesman S Storz G Bacterial small RNA regulators: versatile roles and rapidly evolving variations Cold Spring Harbor perspectives in biology 2011 3 12 10.1101/cshperspect.a003798 PMC3225950 20980440

[B21] Kroger C Dillon SC Cameron AD Papenfort K Sivasankaran SK Hokamp K Chao Y Sittka A Hebrard M Handler K The transcriptional landscape and small RNAs of *Salmonella enterica* serovar Typhimurium Proc Natl Acad Sci USA 2012 109 20 E1277 1286 10.1073/pnas.1201061109 22538806 PMC3356629

[B22] van Heeswijk WC Hoving S Molenaar D Stegeman B Kahn D Westerhoff HV An alternative PII protein in the regulation of glutamine synthetase in *Escherichia coli* Mol Microbiol 1996 21 1 133 146 10.1046/j.1365-2958.1996.6281349.x 8843440

[B23] Genschik P Drabikowski K Filipowicz W Characterization of the *Escherichia coli* RNA 3'-terminal phosphate cyclase and its sigma^54^-regulated operon J Biol Chem 1998 273 39 25516 25526 10.1074/jbc.273.39.25516 9738023

[B24] Huala E Stigter J Ausubel FM The central domain of *Rhizobium leguminosarum* DctD functions independently to activate transcription J Bacteriol 1992 174 4 1428 1431 1735730 10.1128/jb.174.4.1428-1431.1992 PMC206444

[B25] Perkins-Balding D Duval-Valentin G Glasgow AC Excision of IS*492* requires flanking target sequences and results in circle formation in *Pseudoalteromonas atlantica* J Bacteriol 1999 181 16 4937 4948 10438765 10.1128/jb.181.16.4937-4948.1999 PMC93982

[B26] Porwollik S Frye J Florea LD Blackmer F McClelland M A non-redundant microarray of genes for two related bacteria Nucleic Acids Res 2003 31 7 1869 1876 10.1093/nar/gkg298 12655003 PMC152813

[B27] Klose KE Mekalanos JJ Simultaneous prevention of glutamine synthesis and high-affinity transport attenuates *Salmonella typhimurium* virulence Infect Immun 1997 65 2 587 596 9009317 10.1128/iai.65.2.587-596.1997 PMC176100

[B28] Gopel Y Luttmann D Heroven AK Reichenbach B Dersch P Gorke B Common and divergent features in transcriptional control of the homologous small RNAs GlmY and GlmZ in Enterobacteriaceae Nucleic Acids Res 2011 39 4 1294 1309 10.1093/nar/gkq986 20965974 PMC3045617

[B29] Hirschman J Wong PK Sei K Keener J Kustu S Products of nitrogen regulatory genes *ntrA* and *ntrC* of enteric bacteria activate *glnA* transcription in vitro: evidence that the *ntrA* product is a sigma factor Proc Natl Acad Sci USA 1985 82 22 7525 7529 10.1073/pnas.82.22.7525 2999766 PMC390849

[B30] Zimmer DP Soupene E Lee HL Wendisch VF Khodursky AB Peter BJ Bender RA Kustu S Nitrogen regulatory protein C-controlled genes of *Escherichia coli*: scavenging as a defense against nitrogen limitation Proc Natl Acad Sci USA 2000 97 26 14674 14679 10.1073/pnas.97.26.14674 11121068 PMC18977

[B31] Kiupakis AK Reitzer L ArgR-independent induction and ArgR-dependent superinduction of the *astCADBE* operon in *Escherichia coli* J Bacteriol 2002 184 11 2940 2950 10.1128/JB.184.11.2940-2950.2002 12003934 PMC135064

[B32] Riordan JT Tietjen JA Walsh CW Gustafson JE Whittam TS Inactivation of alternative sigma factor 54 (RpoN) leads to increased acid resistance, and alters locus of enterocyte effacement (LEE) expression in Escherichia coli O157: H7 Microbiology 2010 156 Pt 3 719 730 19942657 10.1099/mic.0.032631-0 PMC2889430

[B33] Gardner AM Gessner CR Gardner PR Regulation of the nitric oxide reduction operon (*norRVW*) in *Escherichia coli*. Role of NorR and σ^54^ in the nitric oxide stress response J Biol Chem 2003 278 12 10081 10086 10.1074/jbc.M212462200 12529359

[B34] Maier T Binder U Bock A Analysis of the *hydA* locus of *Escherichia coli*: two genes (*hydN* and *hypF*) involved in formate and hydrogen metabolism Arch Microbiol 1996 165 5 333 341 10.1007/s002030050335 8661925

[B35] Lutz S Bohm R Beier A Bock A Characterization of divergent NtrA-dependent promoters in the anaerobically expressed gene cluster coding for hydrogenase 3 components of *Escherichia coli* Mol Microbiol 1990 4 1 13 20 10.1111/j.1365-2958.1990.tb02010.x 2181234

[B36] Janaszak A Majczak W Nadratowska B Szalewska-Palasz A Konopa G Taylor A A sigma^54^-dependent promoter in the regulatory region of the *Escherichia coli rpoH* gene Microbiology 2007 153 Pt 1 111 123 17185540 10.1099/mic.0.2006/000463-0

[B37] Ramirez-Santos J Collado-Vides J Garcia-Varela M Gomez-Eichelmann MC Conserved regulatory elements of the promoter sequence of the gene *rpoH* of enteric bacteria Nucleic Acids Res 2001 29 2 380 386 10.1093/nar/29.2.380 11139607 PMC29668

[B38] Lloyd LJ Jones SE Jovanovic G Gyaneshwar P Rolfe MD Thompson A Hinton JC Buck M Identification of a new member of the phage shock protein response in *Escherichia coli*, the phage shock protein G (PspG) J Biol Chem 2004 279 53 55707 55714 10.1074/jbc.M408994200 15485810

[B39] Birkmann A Sawers RG Bock A Involvement of the *ntrA* gene product in the anaerobic metabolism of *Escherichia coli* Mol Gen Genet 1987 210 3 535 542 10.1007/BF00327209 3323848

[B40] Miller KA Phillips RS Mrazek J Hoover TR Salmonella utilizes D-glucosaminate via a mannose family phosphotransferase system permease and associated enzymes J Bacteriol 2013 195 18 4057 4066 10.1128/JB.00290-13 23836865 PMC3754740

[B41] Ibanez-Ruiz M Robbe-Saule V Hermant D Labrude S Norel F Identification of RpoS (sigma^S^)-regulated genes in *Salmonella enterica* serovar typhimurium J Bacteriol 2000 182 20 5749 5756 10.1128/JB.182.20.5749-5756.2000 11004173 PMC94696

[B42] Swords WE Cannon BM Benjamin WH Jr Avirulence of LT2 strains of *Salmonella typhimurium* results from a defective *rpoS* gene Infect Immun 1997 65 6 2451 2453 9169789 10.1128/iai.65.6.2451-2453.1997 PMC175341

[B43] Barrangou R Fremaux C Deveau H Richards M Boyaval P Moineau S Romero DA Horvath P CRISPR provides acquired resistance against viruses in prokaryotes Science 2007 315 5819 1709 1712 10.1126/science.1138140 17379808

[B44] Wiedenheft B Zhou K Jinek M Coyle SM Ma W Doudna JA Structural basis for DNase activity of a conserved protein implicated in CRISPR-mediated genome defense Structure 2009 17 6 904 912 10.1016/j.str.2009.03.019 19523907

[B45] Buck M Cannon W Specific binding of the transcription factor σ^54^ to promoter DNA Nature 1992 358 6385 422 424 10.1038/358422a0 1641025

[B46] Mrazek J Finding sequence motifs in prokaryotic genomes–a brief practical guide for a microbiologist Brief Bioinform 2009 10 5 525 536 10.1093/bib/bbp032 19553402

[B47] Crooks GE Hon G Chandonia JM Brenner SE WebLogo: a sequence logo generator Genome Res 2004 14 6 1188 1190 10.1101/gr.849004 15173120 PMC419797

[B48] Doucleff M Pelton JG Lee PS Nixon BT Wemmer DE Structural basis of DNA recognition by the alternative sigma-factor, σ^54^ J Mol Biol 2007 369 4 1070 1078 10.1016/j.jmb.2007.04.019 17481658 PMC2680387

[B49] Cannon W Wigneshweraraj SR Buck M Interactions of regulated and deregulated forms of the sigma^54^ holoenzyme with heteroduplex promoter DNA Nucleic Acids Res 2002 30 4 886 893 10.1093/nar/30.4.886 11842099 PMC100350

[B50] Li GW Elf J Single molecule approaches to transcription factor kinetics in living cells FEBS Lett 2009 583 24 3979 3983 10.1016/j.febslet.2009.11.035 19925790

[B51] Johansson LU Solera D Bernardo LM Moscoso JA Shingler V sigma^54^-RNA polymerase controls sigma^70^-dependent transcription from a non-overlapping divergent promoter Mol Microbiol 2008 70 3 709 723 10.1111/j.1365-2958.2008.06440.x 18786144

[B52] Friedman LJ Gelles J Mechanism of transcription initiation at an activator-dependent promoter defined by single-molecule observation Cell 2012 148 4 679 689 10.1016/j.cell.2012.01.018 22341441 PMC3479156

[B53] Feklistov A Mekler V Jiang Q Westblade LF Irschik H Jansen R Mustaev A Darst SA Ebright RH Rifamycins do not function by allosteric modulation of binding of Mg^2+^ to the RNA polymerase active center Proc Natl Acad Sci USA 2008 105 39 14820 14825 10.1073/pnas.0802822105 18787125 PMC2567451

[B54] Raffaelle M Kanin EI Vogt J Burgess RR Ansari AZ Holoenzyme switching and stochastic release of sigma factors from RNA polymerase in vivo Mol Cell 2005 20 3 357 366 10.1016/j.molcel.2005.10.011 16285918

[B55] Maeda H Fujita N Ishihama A Competition among seven *Escherichia coli* sigma subunits: relative binding affinities to the core RNA polymerase Nucleic Acids Res 2000 28 18 3497 3503 10.1093/nar/28.18.3497 10982868 PMC110723

[B56] Grigorova IL Phleger NJ Mutalik VK Gross CA Insights into transcriptional regulation and sigma competition from an equilibrium model of RNA polymerase binding to DNA Proc Natl Acad Sci USA 2006 103 14 5332 5337 10.1073/pnas.0600828103 16567622 PMC1459355

[B57] Dong T Yu R Schellhorn H Antagonistic regulation of motility and transcriptome expression by RpoN and RpoS in *Escherichia coli* Mol Microbiol 2011 79 2 375 386 10.1111/j.1365-2958.2010.07449.x 21219458

[B58] Chaudhuri RR Yu L Kanji A Perkins TT Gardner PP Choudhary J Maskell DJ Grant AJ Quantitative RNA-seq analysis of the *Campylobacter jejuni* transcriptome Microbiology 2011 157 Pt 10 2922 2932 21816880 10.1099/mic.0.050278-0 PMC3353397

[B59] Damron FH Owings JP Okkotsu Y Varga JJ Schurr JR Goldberg JB Schurr MJ Yu HD Analysis of the *Pseudomonas aeruginosa* regulon controlled by the sensor kinase KinB and sigma factor RpoN J Bacteriol 2012 194 6 1317 1330 10.1128/JB.06105-11 22210761 PMC3294845

[B60] Dasgupta N Wolfgang MC Goodman AL Arora SK Jyot J Lory S Ramphal R A four-tiered transcriptional regulatory circuit controls flagellar biogenesis in *Pseudomonas aeruginosa* Mol Microbiol 2003 50 3 809 824 10.1046/j.1365-2958.2003.03740.x 14617143

[B61] Francke C Groot Kormelink T Hagemeijer Y Overmars L Sluijter V Moezelaar R Siezen RJ Comparative analyses imply that the enigmatic Sigma factor 54 is a central controller of the bacterial exterior BMC Genomics 2011 12 385 10.1186/1471-2164-12-385 21806785 PMC3162934

[B62] Xiao Y Wigneshweraraj SR Weinzierl R Wang YP Buck M Construction and functional analyses of a comprehensive sigma54 site-directed mutant library using alanine-cysteine mutagenesis Nucleic Acids Res 2009 37 13 4482 4497 10.1093/nar/gkp419 19474350 PMC2715252

[B63] Buck M Gallegos MT Studholme DJ Guo Y Gralla JD The bacterial enhancer-dependent sigma(54) (sigma(N)) transcription factor J Bacteriol 2000 182 15 4129 4136 10.1128/JB.182.15.4129-4136.2000 10894718 PMC101881

[B64] Grana D Youderian P Susskind MM Mutations that improve the *ant* promoter of *Salmonella* phage P22 Genetics 1985 110 1 1 16 3996894 10.1093/genetics/110.1.1 PMC1202550

[B65] Kelly MT Hoover TR Mutant forms of *Salmonella typhimurium* sigma^54^ defective in transcription initiation but not promoter binding activity J Bacteriol 1999 181 11 3351 3357 10348845 10.1128/jb.181.11.3351-3357.1999 PMC93800

[B66] Maloy S Stewart VJ Taylor RK Genetic Analysis of Pathogenic Bacteria: A Laboratory Manual 1996 Plainview, NY: Cold Spring Harbor Laboratory Press

[B67] Kustu SG McFarland NC Hui SP Esmon B Ames GF Nitrogen control of *Salmonella typhimurium*: co-regulation of synthesis of glutamine synthetase and amino acid transport systems J Bacteriol 1979 138 1 218 234 35521 10.1128/jb.138.1.218-234.1979 PMC218260

[B68] Hughes KT Youderian P Simon MI Phase variation in *Salmonella*: analysis of Hin recombinase and hix recombination site interaction in vivo Genes Dev 1988 2 8 937 948 10.1101/gad.2.8.937 3049239

[B69] Wang Q Frye JG McClelland M Harshey RM Gene expression patterns during swarming in *Salmonella typhimurium*: genes specific to surface growth and putative new motility and pathogenicity genes Mol Microbiol 2004 52 1 169 187 10.1111/j.1365-2958.2003.03977.x 15049819

[B70] Porwollik S McClelland M Lateral gene transfer in *Salmonella* Microbes Infect 2003 5 11 977 989 10.1016/S1286-4579(03)00186-2 12941390

[B71] Xia XQ McClelland M Porwollik S Song W Cong X Wang Y WebArrayDB: cross-platform microarray data analysis and public data repository Bioinformatics 2009 25 18 2425 2429 10.1093/bioinformatics/btp430 19602526 PMC2735672

[B72] Xia X McClelland M Wang Y WebArray: an online platform for microarray data analysis BMC Bioinforma 2005 6 306 10.1186/1471-2105-6-306 PMC1327694 16371165

[B73] Kelly MT Hoover TR The amino terminus of *Salmonella enterica* serovar Typhimurium sigma^54^ is required for interactions with an enhancer-binding protein and binding to fork junction DNA J Bacteriol 2000 182 2 513 517 10.1128/JB.182.2.513-517.2000 10629201 PMC94304

[B74] Miller JH A Short Course in Bacterial Genetics: A Laboratory Manual and Handbook for Escherichia coli and Related Bacteria 1992 Plainview, NY: Cold Spring Harbor Press

