# Supplementary material for: Use of a promiscuous, constitutively-active bacterial enhancer-binding protein to define the σ54 (RpoN) regulon of Salmonella Typhimurium LT2
Source: BMC Genomics. 2013 Sep 5;14:602. doi: 10.1186/1471-2164-14-602 (PMC3844500; doi:10.1186/1471-2164-14-602)
Supplement: Additional file 1 — Complete list of microarray results. The file contains data for all genes within operons that showed up-regulation by σ54. [file 1471-2164-14-602-S1.pdf]

Additional File 1: Microarray results for WT and Δ*rpoN* *S.* Typhimurium LT2 strains expressing DctD250

Locus Tag[a]	Gene Symbol	Function	WT/Δ*rpoN*[b]
Known σ^{54}-dependent operons and sRNA genes:			
**STM0368**	*prpB*	Proprionate catabolism (putative)	**45**
**STM0369**	*prpC*		**25**
**STM0370**	*prpD*		**72**
**STM0371**	*prpE*		**21**[c]
**STM0830**	*glnH*	Glutamine high-affinity transporter	**7.1**
STM0829	*glnP*		2.4
STM0828	*glnQ*		2.9
**STM2355**	*argT*	Lysine/arginine/ornithine transport	**3.5**
STM_R0152	*tke1*	GlmY sRNA	0.9
STM_R0167	*sraJ*	GlmZ sRNA	1.1
**STM4007**	*glnA*	Glutamine synthetase	**48**
**STM4006**	*glnL*		**3.5**
**STM4005**	*glnG*		**3.6**
Predicted σ^{54}-dependent operons:			
**STM0462**	*glnK*	Nitrogen regulatory protein pII	**3.6**
**STM0463**	*amtB*	Ammonium transport (putative)	**190**
**STM0577**		PTS system (putative)	**67**
**STM0576**			**3.8**
**STM0575**			**3.5**
**STM0574**			**59**
**STM0573**			**18**
**STM0572**			**23**
**STM0649.S**		Hydrolase (putative)	**11**
**STM0650**			**42**
**STM0651**			**8.8**
STM0652			1.2
STM0653	*ybeL*		1.2
STM0665	*gltI*	Glutamate/aspartate transporter	1.8
STM_R0126	*sroC*		0.81
STM0664	*gltJ*		1.3
STM0663	*gltK*		1
STM0662	*gltL*		1.9
STM1285	*yeaG*	Serine protein kinase (putative)	2.5
STM1284	*yeaH*		2.5
STM1303	*astC*	Arginine/ornithine/glutamine metabolism	2.4

Locus Tag[a]	Gene Symbol	Function	WT/Δ*rpoN*[b]
STM1304	*astA*		2.4
STM1305	*astD*		2.6
**STM1306**	*astB*		**3.3**
STM1307	*astE*		2.4
**STM1690**	*pspA*	Phage shock proteins	**17**
STM1689	*pspB*		0.9
**STM1688**	*pspC*		**4**
STM1687	*pspD*		1.3
STM1686	*pspE*		1.5
**STM2360**		Amino acid transport (putative)	**100**
**STM2359**			**11**
**STM2358**			**18**
**STM2357**			**3.3**
STM2356	*ubiX*		1.2
**STM2840**		Nitric oxide reductase	**16**
**STM2841**	*ygbD*		**7.1**
**STM2842**	*hypF*	Hydrogenase maturation proteins	**13**
**STM2843**	*hydN*		**14**
**STM2853**	*hycA*	Hydrogenase 3	**26**
**STM2852**	*hycB*		**12**
**STM2851**	*hycC*		**170**
STM2850	*hycD*		1.9
**STM2849**	*hycE*		**96**
**STM2848**	*hycF*		**25**
**STM2847**	*hycG*		**30**
**STM2846**	*hycH*		**6.4**
**STM2845**	*hycI*		**12**
**STM2844**			**9.4**
**STM2854**	*hypA*	Formate-hydrogen lyase system	**5.6**
**STM2855**	*hypB*		**39**
**STM2856**	*hypC*		**6.3**
**STM2857**	*hypD*		**25**
**STM2858**	*hypE*		**31**
**STM3521**		RNA repair system (putative)	**71**
**STM3519**	*rtcB*		**27**
**STM3518**	*rtcA*		**4.3**
STM3568	*rpoH*	Heat shock sigma factor (σ^{32})	1.7
**STM3772**		PTS system (putative)	**39**
**STM3771**			**37**
**STM3770**			**60**

Locus Tag[a]	Gene Symbol	Function	WT/Δ*rpoN*[b]
**STM3769.S**			**31**
**STM3768**			**24**
**STM3767**			**41**
STM3766			1.4
**STM4172**	*zraP*	Zinc resistance-associated protein	**16**
**STM4173**	*hydH*	Zinc resistance two-component system	**3.7**
STM4174	*hydG*		2.8
STM4244	*pspG*	Phage shock protein	1.4
**STM4285**	*fdhF*	Formate dehydrogenase	**29**
**STM4535**		PTS system (putative)	**16**
STM4536			1.7
**STM4537**			**66**
**STM4538**			**13**
**STM4539**			**43**
**STM4540.S**			**25**
Novel σ[54]-dependent operon:			
STM2944	*ygcB*	CRISPR-associated proteins	1.6
STM2943			1.2
STM2942			1.3
STM2941	*yghJ*		1.2
STM2940			1.4
STM2939	*ygcH*		2.8
**STM2938**			**4.1**
STM2937	*ygbF*		0.8
Possible indirectly regulated σ[54]-dependent operons:			
STM0144	*ppdD*	Pilin biogenesis (putative)	0.78
**STM0143**	*hofB*		**3.3**
STM0142	*hofC*		2.2
STM0515	*allA*	Allantoin utilization	1.1
STM0516	*allR*		1
STM0517	*gcl*		2
STM0518	*gip*		1.6
STM0519	*glxR*		2
STM0520			1.8
STM0521	*ybbV*		1.3
**STM0523**	*allB*		**3.2**
STM0524	*ybbY*		1.4
STM0525	*glxK*		0.99
**STM0791**	*hutH*	Histidine-ammonia lyase	**3.8**

Locus Tag[a]	Gene Symbol	Function	WT/Δ*rpoN*[b]
**STM1252**		Cytoplasmic protein (putative)	**3.1**
STM2038	*pduA*	Propanediol utilization	0.75
STM2039	*pudB*		1.1
STM2040	*pduC*		2
STM2041	*pduD*		1.5
STM2042	*pduE*		0.46
**STM2043**	*pduG*		**3.3**
STM2044	*pduH*		0.46
STM2045	*pduJ*		0.58
STM2046	*pduK*		0.7
STM2047	*pduL*		4.3[c]
STM2048	*pduM*		2.5
STM2049	*pduN*		0.87
STM2050	*pduO*		1.4
STM2051	*pduP*		1.4
STM2052	*pduQ*		1.5
STM2053	*pduS*		1.7
STM2054	*pduT*		1.8
STM2055	*pduU*		0.94
STM2056	*pduV*		0.61
STM2057	*pduW*		2.1
STM2058	*pduX*		1.6
STM2149	*stcD*	Chaperone-usher fimbriae (putative)	1.5
**STM2150**	*stcC*		**3.2**
STM2151	*stcB*		1.9
STM2152	*stcA*		0.82
**STM2572**	*yfhH*	Membrane transport protein (putative)	**3.1**
STM2773	*iroB*	Siderophore production	2.1
STM2774	*iroC*		0.95
**STM2775**	*iroD*		**4**
STM2776	*iroE*		1.2
**STM2839**	*ygaA*	EBP activator for STM2840 operon (putative)	**3.2**
**STM2859**	*fhlA*	EBP activator for *fdhF/hyc/hyp* operons	**3.5**
STM2899	*invF*	Cell invasion proteins	2
**STM2898**	*invG*		**5.9**
STM2897	*invE*		1.7
STM2896	*invA*		1.8
STM2895	*invB*		1
STM2894	*invC*		1.9
STM2893	*invI*		0.9
STM2892	*invJ*		2
STM2891	*spaO*		1.9

Locus Tag[a]	Gene Symbol	Function	WT/Δ*rpoN*[b]
STM2890	*spaP*		1.6
STM2889	*spaQ*		0.69
STM2888	*spaR*		2.7
STM2887	*spaS*		1.4
STM2886	*sicA*		0.71
STM2885	*sipB*		1.1
STM2884	*sipC*		1.1
STM2883	*sipD*		1.2
STM2882	*sipA*		1.7
STM3253		PTS system/sugar metabolism (putative)	1.5
STM3254			1.8
STM3255			1
**STM3256**			**3.7**
STM3257			2.9
STM3258			1.4
STM3259			1.1
STM3260			2
STM3261			1.3
STM3262			1

[a] Locus tags for all genes within operons in which at least one gene was up-regulated ≥3-fold (p-value <0.02) by RpoN in the microarray analysis; up-regulated genes are in **black** type and genes below the 3-fold cut off are in **grey** type). The up-regulated operons are grouped as known, predicted, novel, or possible indirectly regulated, as defined in Results.
[b]Both WT and Δ*rpoN* strains contain pPBHP92 expressing DctD250. Signal ratios below the 3-fold cutoff for up-regulation are displayed in **grey**.
[c]above 3-fold cutoff, but p-value >0.02
