# Supplementary material for: Use of a promiscuous, constitutively-active bacterial enhancer-binding protein to define the σ54 (RpoN) regulon of Salmonella Typhimurium LT2
Source: BMC Genomics. 2013 Sep 5;14:602. doi: 10.1186/1471-2164-14-602 (PMC3844500; doi:10.1186/1471-2164-14-602)
Supplement: Additional file 2 — Sequences used to generate Position-Specific Scoring Matrix. [file 1471-2164-14-602-S2.pdf]

Additional File 2: Sequences used to generate the Position-Specific Score Matrix (PSSM)

Locus	Gene	Sequence
STM0368	*prpB*	TGGCATAGCCTTTGCTTT
STM0462	*glnK*	TGGCACATCCTTTGCAAT
STM0577		TGGCACGCCGTTTGCCAT
STM0649.S		TGGCACGCCTTTTGATTA
STM0665	*gltI*	TGGCACGTCTATTGCTTT
STM0830	*glnH*	TGGCATGATTTTTTCATT
STM1285	*yeaG*	TGGCATGAGAGTTGCTTT
STM1303	*argD*	TGGCACGAATGCTGCAAT
STM1690	*pspA*	TGGCACGCAAATTGTATT
STM2354*	*hisJ*	TGGCACGATAGTCGCATC
STM2355	*argT*	TGGCATAAGACCTGCATG
STM2360		TGGCATGCCTTTTGCTTT
STM_R0152	*glmY*	TGGCACAATTACTGCATA
STM2840		TGGCACACTAGCTGCAAT
STM2843	*hydN*	TGGCACGATTCGTGTATA
STM2853	*hycA*	TGGCATGGAAAATGCTTA
STM2854	*hypA*	TGGCATAAATATTGCTTT
STM3521		TGGCACGCTGGTTGCAAT
STM3568	*rpoH*	TGGCACGGTTGTTGCTCG
STM3772		TGGCACAACCTTTGCTCT
STM_R0167	*glmZ*	TGGCACGTTATGTGCAAT
STM4007	*glnA*	TGGCACAGATTTCGCTTT
STM4172	*zraP*	TGGCACGGAAGATGCAAG
STM4173	*hydH*	TGGCATGATCTCTGCTTA
STM4244	*pspG*	TGGCATGATTTTTGTAAG
STM4285	*fdhF*	TGGCATAAAACATGCATA
STM4535		TGGCACGCCGCTTGCTCT

*Although this sequence has been predicted to be a σ^{54}-dependent promoter, there is evidence indicating that this is not an active promoter [2].
