# Supplementary material for: Use of a promiscuous, constitutively-active bacterial enhancer-binding protein to define the σ54 (RpoN) regulon of Salmonella Typhimurium LT2
Source: BMC Genomics. 2013 Sep 5;14:602. doi: 10.1186/1471-2164-14-602 (PMC3844500; doi:10.1186/1471-2164-14-602)
Supplement: Additional file 3 — Oligonucleotides used. [file 1471-2164-14-602-S3.pdf]

**Additional File 3: Oligonucleotides used in this study**

| Name | Forward | Reverse |
|---|---|---|
| pDS11MCS | CTAGAATTGAGCTCATTGGTACCATTG | GATCCAATGGTACCAATGAGCTCAATT |
| pDS12MCS | AGCTTAGATCTCTAGAGTCGACGGTACCATTG | GATCCAATGGTACCGTCGACTCTAGAGATCTA |
| LM-PCR linker | AGAAGCTTGAATTCGAGCAGTCAG | CTGCTCGAATTCAAGCTTCT |
| DctD | ATTGGATCCCACTCGACCGGAATTATCG | TTTTATCAGACCGCTTCTGC |
| STM0224 (*yaeT*) | GACATGAGTCCTTAGTCCG | AGGTAACATTACGCTATGGG |
| STM0334 | CGATAGCGGAAACAAAACCG | TCAGGAAGCGAATATCTGGG |
| STM0462 (*glnK*) | TCGCCCATCATGCACCGTCG | TCCCTGAATGCCAATGGAAG |
| STM0504 (*ybbN*) | AGGGCGATGTCCTAGTCC | AGGACCTTCCAAAACGCG |
| STM0699 | CTCTGCCCGTATGTTGTCCC | ATACGCACCACGCAAACCG |
| STM0961 (*lolA*) | GGTAGCCTGTTCTACAAACGG | GGAGTGTCGAAACCTGAGG |
| STM1285 (*yeaG)* | GTAGAGGCTCCCGGAAGAGG | ATCCACCAGCCTTTTCTACC |
| STM1586 | GCGTTTTACCGTCTGCCG | GTAAGAAGTTGCTGGATGACGG |
| STM1594 | CTCTGGATGTACTCGACGG | AGATACTCAAAACTACGCAGCG |
| STM1697 | GGAAATAACGTGCCCTGGG | ATCTGTTGCGGACAATCGC |
| STM2016 (*cobT*) | TGGCTTTCTTTCCTACTCGG | GGCAGGACAATATTACTGGC |
| STM2430 (*cysK*) | AGCTGCAGGAAGATGAAAGC | GAATCAATGCCAGGTGAGG |
| STM_R0152 (*glmY)* | AAGGGGCTGACATAAGAAGG | TTAGGTGTTGCAGGTGTTGC |
| STM2939 | TGAACACGATGGCTTAACGG | CATCAGCAAATCGTGACGC |
| STM2957 (*rumA*) | CGTCAATGTCGAACAGTGCC | CGACCATTTGCTGGTTTACCG |
| STM3127 | GGACTGTTTTCATCGACCC | GGTAAATAACGTTGTGACGC |
| STM3521 | AAGCGTAGAATCTAAAGGAAG | AGGGAATTGTCGCTTGCC |
| STM3568 (*rpoH*) | CAAATCCTCTCAATCAGTATTGC | GTTCGCAGGGAAAGAGTCC |
| STM_R0167 (*glmZ*) | TTCTGTCTCCACCGGGCGA | TCCAGGGTGTTTGATGAGG |
| STM4007 | GCGCGTTATTGTACACGG | TGTACTCTCCCGGATTGG |
